# Aquatic Risks at the Landscape Scale: A Case Study for Pyrethroid Use in Pome Fruit Orchards in Belgium

**DOI:** 10.1021/acs.est.3c02716

**Published:** 2023-10-05

**Authors:** Willem B. Buddendorf, Louise Wipfler, Wim Beltman, Hans Baveco, Maarten C. Braakhekke, Sascha Bub, André Gergs, Thorsten Schad

**Affiliations:** † Wageningen Environmental Research, P.O. Box 47, 6700AA Wageningen, The Netherlands; ‡ iES Landau, Institute for Environmental Sciences, University of Kaiserslautern-Landau (RPTU), Fortstraße 7, D-76829 Landau, Germany; § Research & Development, Crop Science, Environmental Modelling, Bayer AG, 40789 Monheim, Germany

**Keywords:** environmental risk assessment, landscape scale, spatially explicit, temporally explicit, integrated fate and effect modeling

## Abstract

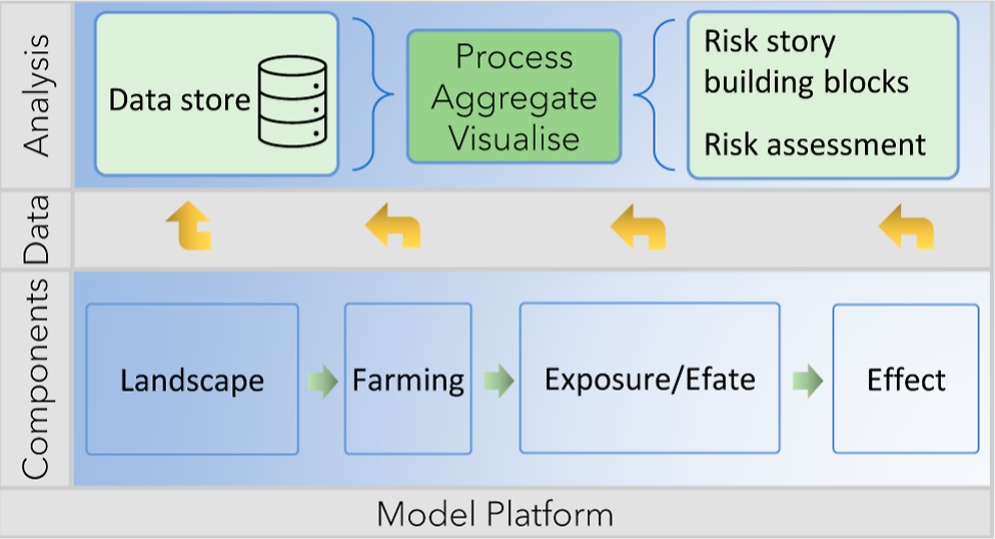

Procedures for environmental risk assessment for pesticides are under continuous development and subject to debate, especially at higher tier levels. Spatiotemporal dynamics of both pesticide exposure and effects at the landscape scale are largely ignored, which is a major flaw of the current risk assessment system. Furthermore, concrete guidance on risk assessment at landscape scales in the regulatory context is lacking. In this regard, we present an integrated modular simulation model system that includes spatiotemporally explicit simulation of pesticide application, fate, and effects on aquatic organisms. As a case study, the landscape model was applied to the Rummen, a river catchment in Belgium with a high density of pome fruit orchards. The application of a pyrethroid to pome fruit and the corresponding drift deposition on surface water and fate dynamics were simulated. Risk to aquatic organisms was quantified using a toxicokinetic/toxicodynamic model for individual survival at different levels of spatial aggregation, ranging from the catchment scale to individual stream segments. Although the derivation of landscape-scale risk assessment end points from model outputs is straightforward, a dialogue within the community, building on concrete examples as provided by this case study, is urgently needed in order to decide on the appropriate end points and on the definition of representative landscape scenarios for use in risk assessment.

## Abstract 2

Using an integrated environmental fate and effect model, concrete examples are presented, which can inform a discussion on the landscape-scale risk assessment framework.

## 1 Introduction

The reliability and relevance of environmental risk assessment (RA) within registration procedures are currently challenged based on the evidence of ecological impacts of pesticide uses.^1,2^ Environmental RA follows a tiered approach; a key principle is that lower tiers of the RA are more conservative than higher tiers that aim to be more realistic.^3,4^ Regardless of the RA tier, all tiers should address the same protection and therefore need to be protective, internally consistent, and cost-effective, and finally, they should address the problem with increasing accuracy and precision when moving from lower to higher tiers.^5^ The disregard of the spatiotemporal dynamics of pesticide exposure and of the spatiotemporally variable behavior of organisms is seen as an important flaw of the current RA system.^6,7^ For instance, the (sensitive) life stages of the species tested under laboratory conditions for tier 1 RA may or may not co-occur in space or time with ecotoxicologically relevant concentrations under field conditions. Most importantly, laboratory studies typically impose a constant exposure regime and optimal environmental conditions to avoid any confounding effects in the derivation of effect thresholds. Subsequently, such effect thresholds are compared to exposure simulated by, e.g., FOCUS surface water scenarios.^8,9^ These static FOCUS surface water scenarios are intended to provide a realistic worst-case exposure profile over time that represents single edge-of-field ponds, ditches, and streams with strongly simplified upstream catchments. In practice, the outcome of fate and effect processes in a heterogeneous landscape will depend on local variations in, e.g., landscape configuration, farming practices, and connectivity between streams. Ignoring these factors may lead to an unrealistic representation of the spatiotemporal pattern of exposure and the potential effects. It is not feasible to experimentally test such effect dynamics across the range of possible conditions, particularly at landscape levels. However, mechanistic modeling approaches allow us to incorporate temporal exposure dynamics and translate these to effect estimates. For example, toxicokinetic/toxicodynamic (TKTD) models, such as the General Unified Threshold Model of Survival^10^ (GUTS), are suggested to support the environmental RA process by predicting the potential effects of time-variable exposures^11,12^ as part of the tier 2C exposure refinement option in Europe. Embedding TKTD models in a spatial approach, e.g., applying these individual-level models at many locations in a landscape (i.e., moving to the highest tier, tier 4, in the RA scheme) allows for spatially heterogeneous exposure dynamics.

In recognition of spatial and temporal variability, more realism is included by assessing the risk at the landscape level. Indeed, the European Food and Safety Authority Panel on Plant Protection Products and their Residues (EFSA PPR henceforth)^13^ identifies landscape-scale model application as the highest tier for the aquatic RA, i.e., with the highest level of realism while addressing the same specific protection goals. Concrete guidance on how to link the outcomes of ecological models to the well-established assessment factors in the tiered aquatic RA, using the margin of safety concept, is given in the EFSA PPR Panel.^12^ The EFSA PPR Panel^5^ proposes the specific protection goal for aquatic invertebrates to be based on their abundance and/or biomass in edge-of-field surface waters. Previous studies have developed approaches to move up toward a landscape scale RA in aquatic systems.^14^ Yet, no guidance currently exists on how to assess environmental risks at the landscape scale in the regulatory context. A framework is needed to link landscape-scale assessment end points to specific protection goals. To further develop such a framework and to enable the development of a common language among stakeholders, example studies with concrete model outcomes are extremely helpful, as we demonstrate in this study. Here, we present an example for aquatic organisms, with the final aim to feed into the discussion of the landscape-scale RA framework and the use of landscape-scale models herein.

For the example study, an integrated model system was developed that includes spatiotemporally explicit simulations of pesticide application, fate, and effects. Here, the landscape scale is defined as a single catchment. Application of the model to the Rummen catchment area in Belgium with 21% of its area covered by fruit orchards is presented. Annual spray application of a pesticide in orchards, subsequent drift deposition to streams, and consequent spatiotemporal fate dynamics are simulated. The risk to aquatic nontarget organisms is then quantified, and landscape scale risk characterization end points are proposed.

## 2 Materials and Methods

### 2.1 Landscape Integrated Fate and Effect Model, xAquaticRisk

Simulations were conducted with the integrated, modular model system xAquaticRisk (v2.67 was used in this paper, available at https://github.com/xlandscape/xAquaticRisk/tree/2.67). The xAquaticRisk model is designed as a modular model system (see the Supporting Information for a schematic overview), and the core model system handles all I/O processes between components and the binary data store (i.e., there is no direct exchange between model components). Each model component can in principle be run as a stand-alone component, provided the required input data are available in the data store. Through the component interface, individual components can be written in a multitude of programming languages, making the xAquaticRisk model highly flexible and versatile. xAquaticRisk considers a network of watercourses in a landscape in which pesticide is applied within specified time windows on agricultural fields. The pesticide enters the water courses via spray drift deposition. Components are simulated in a consecutive order: the pesticide application on the fields, drift inputs, pesticide fate, and effects on aquatic organisms. Each component is briefly described here; more information on used data sets and model inputs and parametrization can be found on the xAquaticRisk GitHub page and in publications of separate model components (see below). Individual components may operate on different scales; for instance, spray drift deposition is simulated per square meter, whereas here, the river network is divided into discrete reaches and environmental fate is simulated per stream reach and hour. Effects are expressed per reach and year, or per reach and day depending on the effect module that is used. The model system handles the technical transitions between scales automatically.

Hourly water depth and discharge per reach were calculated separately (and used as environmental input data to the xAquaticRisk modeling framework) with a hydrological modeling approach,^15^ which was implemented using the catchment modeling framework CMF.^16^ CMF simulates water flows using the catchment area yield approach, which allows for simulating stream processes across the network based on observed discharge data from downstream outflow points (e.g., the catchment outlet or flow gauges near confluences). Cross sections of the reaches were considered to be trapezium-shaped, and Strahler orders were assigned to reaches. Special attention was given to the prediction of water depth and flow velocity, which are important for pesticide concentrations in reaches that receive input from drift.^17^

The agricultural management component generates input data for the model on application characteristics with regard to, e.g., location, timing, application rate, and used equipment. The spray drift component^18,19^ simulates the spray drift deposition per square meter along field edges per day. For each orchard, the application date is picked randomly within a user-defined application window. On the selected date, spray application is assumed to occur at noon. The module simulates variable wind conditions through random draws from the wind-rose and is calibrated on experimental data from Rautmann, Streloke, and Winkler.^20^ Reaches receive spray drift deposition according to the mean rate of deposition (mass per area) of their assumed spray drift surface when they are located downwind from an orchard on the application day.^19^ For each combination of application event, orchard, and day, a random wind direction is assigned (for a given day) based on a draw from a distribution along the wind rose.

Spatiotemporally explicit concentrations in water in the reaches are simulated using the pesticide fate module CASCADE_TOXSWA.^21^ This model simulates pesticide fate in interconnected water courses with variable water flows and water depths. Its kernel is TOXSWA, a pesticide fate model for water and sediment systems developed for single water bodies.^22,23^ The TOXSWA model is used in the registration procedure of plant protection products at a national level in The Netherlands as well as at the EU level.^8,9^ CASCADE_TOXSWA simulates pesticide fate in the reaches based on descriptions of: sorption to organic matter in suspended solids and sediment, transformation in water and sediment, volatilization from water to air, and exchange between water and sediment by diffusion. Briefly, after being deposited on the water surface, the substance dissolves and mixes over the entire reach length. The substance enters the sediment pore water via diffusion and adsorbs to the sediment organic matter. The substance in the water layer (dissolved and adsorbed to suspended solids) is transported due to convection to downstream reaches. After a drift deposition event, the substance flows out of the reach quickly, and concentration time profiles are peaky and short. Uncontaminated upstream water invokes the diffusion of the substance slowly back from the sediment into the water layer. Additionally, degradation takes place in the sediment and water layers, albeit at different rates (see Table S2 in the Supporting Information).

For this model application, the individual reaches are simulated in a bucket-type approach.^21^ Spatially and temporally distributed water depth and flow velocity, drift deposition per reach, and pesticide properties are used as input by the module to calculate hourly average pesticide concentrations in water and sediment.

Hourly averaged pesticide concentrations in water are defined as the relevant exposure concentrations for this study. The effect module LGUTS simulates individual-level effects of exposure to a pesticide in the water. The model performs calculations per reach, per hour, and per species by applying a TKTD model. The applied TKTD model is the “reduced GUTS” model,^10,24^ which is recommended in favor of a full GUTS model when body residue data are not available (as is often the case).^10^ The reduced GUTS model has two versions: the stochastic death (SD) and the individual tolerance (IT) version.^10,24^ These two toxicodynamic assumptions represent two extreme views on survival with different implications for repeated exposures. With SD, the survival probability of an organism decreases whenever the dose metric exceeds a threshold for survival; the hazard rate increases linearly with the scaled damage above the threshold. In contrast, with IT, the threshold follows a frequency distribution within a population, and death is instantaneous when the scaled damage exceeds the individual threshold of an organism. Within a real RA application, the most suitable model version is selected based on model performance criteria.^12^ However, owing to the nature of this study (i.e., a proof of concept case study), here we simply show results for the IT model and present results for the SD model in the Supporting Information for completeness.

The set of GUTS parameters is species- and chemical-specific. GUTS models were previously calibrated and validated for a pyrethroid and three aquatic species *Asellus aquaticus*, *Cloeon dipterum,* and *Gammarus pulex*; for specific parameter values, see Table S1 and Figures S2–S7 in the Supporting Information. The model simulates an arbitrary period of a year from 1st of January to 31st of December. The outcome of the simulation is the survival over time. Here, we focus on the cumulative mortality at the end of each year. A margin of safety approach is applied, in which all simulations are run for a series of multiplication factors applied to the hourly concentration values. From these simulations, LP_50_ (lethal profile 50) values are determined for each reach and year, i.e., the concentration multiplication factor leading to a 50% reduction of survival at the end of the year in the GUTS model. For further details on the approach, see the EFSA PPR Panel.^12^

A model run of 26 years was done with a pyrethroid applied in fruit orchards between April 20 and 30 (differing per orchard and per year) in the study area. The applied dose was 12.5 g/ha of active ingredient. Mitigation options were set, assuming the use of drift-reducing technology resulting in 75% drift reduction on the water courses and a 10 m buffer zone along the surface water. These are realistic but not extreme values, chosen to ensure that some level of effects was seen and landscape-level risks could be characterized. Common for a pyrethroid, the substance has a high sorption coefficient for organic carbon (*K*_oc_) of 1,024,000 L/kg; the transformation half-life is 1000 and 43.9 d in water and sediment, respectively (other substance properties are listed in Table S2 in the Supporting Information). In line with FOCUS scenarios, a warming period of 6 years was applied to the model, followed by a period of 20 years for which the calculated concentrations were used in subsequent modules and analyses.

### 2.2 Case-Study Area

#### 2.2.1 General Description of the Rummen Catchment Area

**Figure 1:**
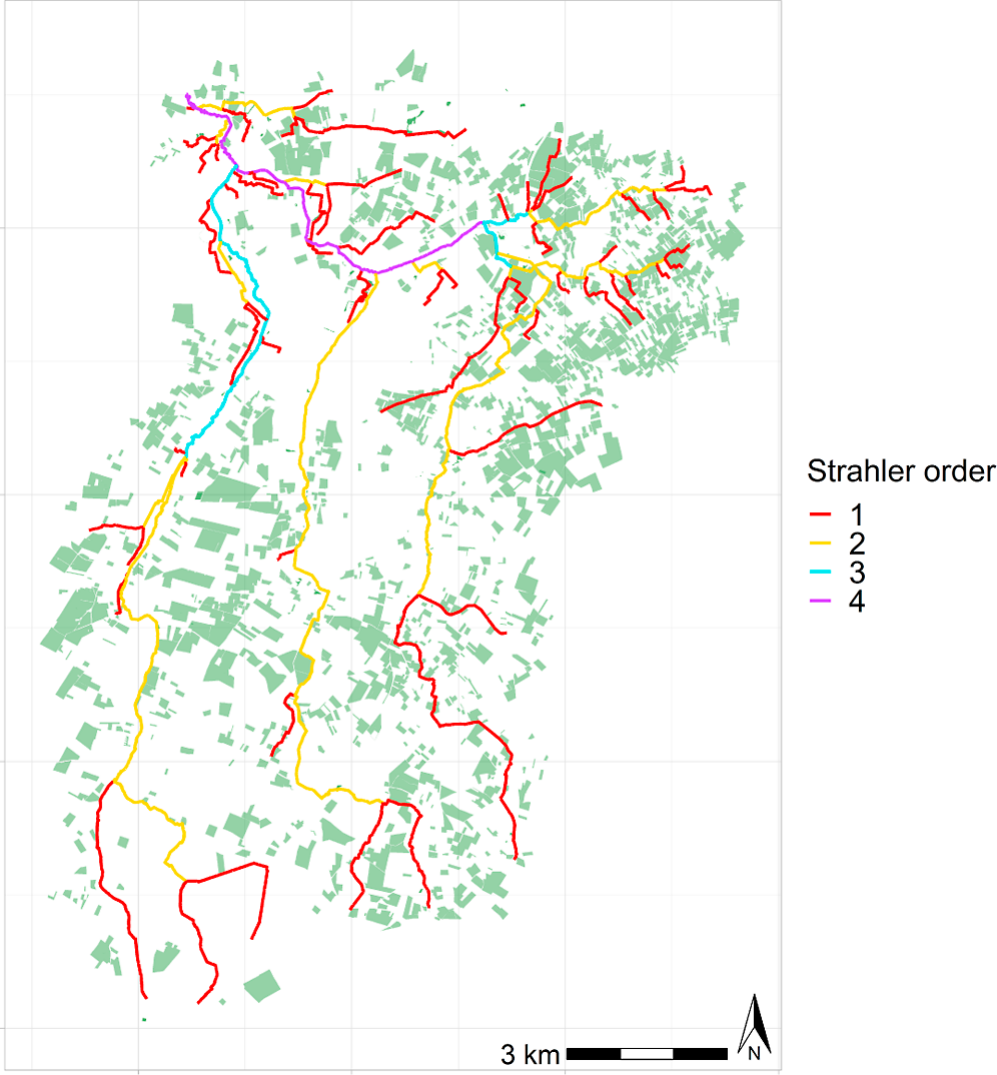
Strahler order of reaches in the Rummen catchment. Green areas represent fruit orchards. The outlet is located in the northwest of the catchment.

The Rummen catchment area (more specifically the Rummen–Melsterbeek catchment in Wallonia in the eastern part of Belgium) is characterized by loamy soils.^25^ It has a drainage area of 150 km^2^ and an average local slope of 2.47%. The total stream length is 146 km, consisting of three major branches (>15 km) and three minor tributaries (<7 km). Headwaters make up a large portion (55%) of the river network. In the catchment, 21% of the area (30.9 km^2^) is covered with fruit orchards, mainly pome (Figure 1). In these orchards, pesticides are applied regularly to control pests and diseases, such as apple scab or fire blight.

### 2.3 Landscape Scenario

For the study, a single landscape scenario was created, consisting of (i) a geo data set containing the river network which was divided into 1708 reaches characterized by median length (100 m, with exceptional cases with different lengths with a minimum of 5 m and a maximum of 110 m, in both cases owing to the practicalities of dividing up the river network), depth, width, bank slope, bottom width, and Manning’s *n*; (ii) a hydrology data set (see Section 2.1) covering the period 1992–2017; (iii) a geo data set containing the locations, shapes, and sizes of orchards; and (iv) daily temperature data (average of preceding 3 days) covering the period 1992–2017 (see https://agri4cast.jrc.ec.europa.eu/dataportal/). The Strahler order was used to set properties (other than length) indicated in (i). An overview of the Rummen catchment, with the locations of the orchards and river network, is shown in Figure 1. Hydrogeographic statistics specific to the Rummen catchment are presented in Table S3 and Figure S7 in the Supporting Information.

For the period 20–30 April, during the full 20 year period, water depths range between 0.01 and 0.5 m across all reaches. Residence times were calculated for each hour by dividing the (dynamic) water volume in the reach by the hourly discharge. This median residence time is longest in Strahler order 1 reaches, 10.3 min, and shortest in Strahler order 4 reaches, 2.6 min (Figure S8). For Strahler order 4 reaches, all residence times are less than 7.6 min. The longest residence times are found in Strahler order 1 reaches, but rarely are they longer than 120 min. The range of maximum deposition values is between 0 and 2162 μg. However, the median deposition value is 0 μg (i.e., no deposition in at least 50% of cases), thus indicating a distribution that is skewed toward lower deposition values (Figure S8).

## 3 Results and Discussion

Below we first present some basic reporting elements of the landscape model (Sections 3.1 and 3.2) that comprehensively report on exposure and effects. Next, a first set of three approaches to landscape-scale RA is presented. The approach can be based on: (i) deriving simple end points where space and time are aggregated at the catchment level (Section 3.3); (ii) deriving end points that consider time and space at local scales by aggregating at the stream segment level (Section 3.4); and (iii) a further elaboration of (ii), aiming to perform a local RA while using (ii) and allowing for an investigation of potential mitigation options (Section 3.5). Links to the current approaches to RA (Section 3.6) as well as limitations of the current model and future developments (Section 3.7) are discussed.

### 3.1 Spatial Differences in Residence Times in the Period of Application

**Figure 2:**
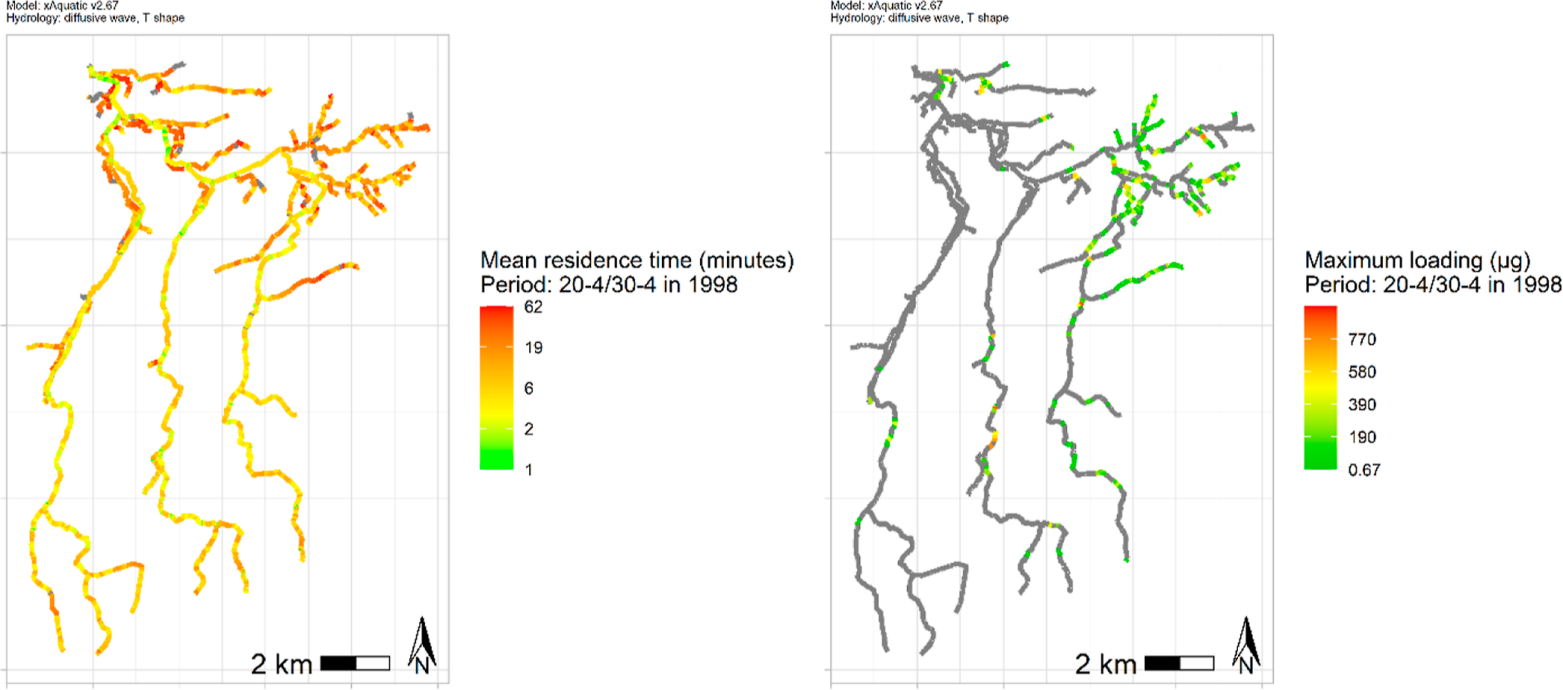
Mean residence time of water in the period 20–30 April 1998 per reach (left) and maximum spray drift deposition μg per reach in the same period (right) for the reaches in the Rummen catchment.

The slower water moves through the system, the longer individuals will be exposed. Hence, the residence time potentially has a strong influence on the occurrence and magnitude of effects. Figure 2 (left) gives an example of the mean residence time in the period of application 20–30 April in the year l998. It shows that mean residence times in this period are ca. 1 h for all reaches. In the southwest part of the catchment, residence times tend to be shorter compared to the northeast part of the catchment, and higher order reaches toward the outlet show shorter residence times.

### 3.2 Spatial Differences in Spray Drift Deposition

The simulated application of pyrethroid in pome fruit orchards in the Rummen catchment resulted in a total mean annual drift deposition of 62.7 mg over a length of 52 km (mean over the 20 year assessment period). Figure 2 (right) shows example drift depositions for the year 1998; drift depositions in exposed reaches range from 0.031 μg up to 1244.6 μg in the northeast, close to the outlet, and in a band in the three main tributaries halfway between the north and south part of the catchment. These main input areas are due to the presence of orchards in close vicinity to the reaches (Figure 1). In the southwest, there are only few orchards directly adjacent to reaches; hence, there is little spray drift deposition. Some of the reaches did not receive any substance at all, i.e., neither by drift deposition nor via transport from upstream reaches. For example, in Strahler order 1 reaches, 38% did not receive substance; yet for Strahler order 2 streams, this is reduced to 1.6%, and in Strahler order 3 and 4 reaches, 89.7 and 87.5% receive substance from transfer alone. Table S4 provides an overview of the percentage of reaches that receive input from both drift and upstream transfer and from upstream transfer only.

### 3.3 Spatiotemporal Landscape-Scale Fate and Effects

**Figure 3:**
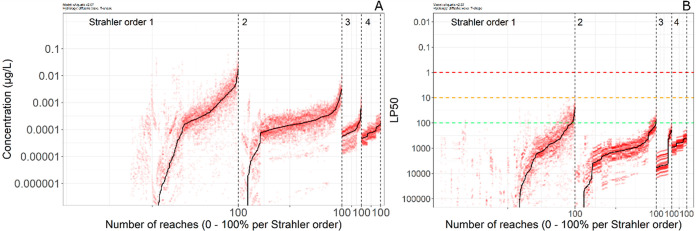
Predicted environmental concentrations (A) and LP_50_ values (B). Red dots are the 20 maximum hourly averaged concentrations per reach per year (A) or 20 LP_50_ values per reach per year for *A. aquaticus* for the GUTS-IT model (B). Reaches are sorted along the *x*-axis by their medians and per Strahler order. The black line shows the median value per reach over 20 assessment years. Concentration values were cut off at 10^–6^ μg/L; LP_50_ values were cut off at a value of 10^5^. Reaches for which the median falls below the cutoff have negligible median exposure/risk but may have individual years with relatively high exposure/risk. Note that the values on the *y*-axes are on a log-scale and are in reverse order for the LP_50_ plot.

In Figure 3A, the yearly maximum of hourly averaged concentrations per reach and per Strahler order is shown. Concentrations tend to be higher for lower Strahler order reaches. Moreover, year-to-year variation is much higher in lower Strahler order reaches than in higher Strahler order reaches, owing to dilution and mixing in the latter, which leads to a reduction and attenuation of fluctuations in concentrations. For assessing exposure in the RA, the exposure assessment goal is proposed as the overall 90th percentile of annual peak concentrations in water, considering all spatial units in the area of use.^3,5^ This is termed the predicted environmental concentration (PEC or the PEC_max_). In this study, the entire concentration profile is available for all simulated reaches and over the simulation period at hourly timescales. These spatiotemporally explicit concentration profiles can be used for effect modeling directly.

Next, the resulting effects on the organisms can be assessed by using the output from the LGUTS module. LP_50_ values were calculated per reach and per year, for the IT and SD versions. An LP_50_ value of 1 indicates a 50% reduced survival rate, which is associated with a high risk. A multiplication factor of 100 is associated with an acceptable risk at tier 2C.^12^ Some Strahler order 1 and 2 reaches have no exposure (see Table S4); for these reaches, the LP_50_ value cannot be calculated and is set to infinite. The ranges of calculated LP_50_ values per Strahler order are provided in Table S5. The results for the GUTS-IT model for *A. aquaticus* are presented here; results for *C. dipterum* and *G. pulex* as well as GUTS-SD results can be found in the Supporting Information (Figures S9 and S10 and Table S6). Figure 3B shows the LP_50_ values over the 20 year assessment period in the Rummen catchment, arranged by the Strahler order and sorted by their median value. LP_50_ values are lowest for reaches of Strahler order 1 and increase with increasing Strahler order. At Strahler orders 3 and 4, there is a series of lines to be seen in the graph (red dots). These lines belong to one specific year each and are a consequence of the configuration of the Rummen catchment. In the Rummen catchment, Strahler orders 3 and 4 reaches have fewer reaches with direct deposition on the reach and the large majority of reaches receive substance via transfer only (Table S4). Consequently, a concentration pattern that starts upstream due to a drift deposition event is also seen in downstream reaches, which causes a uniform pattern of exposure and thus a uniform pattern of effects along these downstream reaches. Conversely, the reaches of Strahler order 1 are less organized (they are situated in different tributaries and different branches) and located in upstream areas, with more reaches receiving direct deposition. In terms of general patterns, these are similar to those for the predicted environmental concentrations: higher variability in lower Strahler order streams and an overall decrease in effects with increasing Strahler order. Again, this is primarily caused by mixing and dilution processes when moving from lower to higher Strahler order streams.

The information presented in Figure 3 gives a good overview of the variation within reaches over time and space and provides a first insight into the role of higher levels of organization like the Strahler order. End points like an overall 90th percentile in space and time for the PECs and an overall 10th percentile in space and time for the LP_50_ are readily calculated, and their interpretation is straightforward. However, by aggregating the available data to such a high level, a lot of information on local fate and effect dynamics becomes implicit. For example, from the plot it is impossible to assess what percentage of space (reaches) is above or below a certain PEC or LP_50_ value for what percentage of time. Yet, this is considered valuable information in the RA context. Therefore, the next step of analysis is aimed at a lower level of aggregation where reaches are considered explicitly in their spatiotemporal presentation.

### 3.4 Explicit Spatiotemporal Landscape-Scale Risk Assessment

Risk managers need to decide upon acceptable levels of risk, which in a spatially and temporally explicit RA framework may consist of defining a range of PEC values or LP_50_ values, each combined with a maximum allowed exceedance frequency or percentage in space and time. Higher PEC values would have a lower allowed frequency (or percentage) of exceedance for space and time compared to lower PEC values; after all, with lower PEC values, there is typically a lower risk. For effects, low LP_50_ values would have lower allowed frequency (percentages) of space and time than higher LP_50_ values.

**Figure 4:**
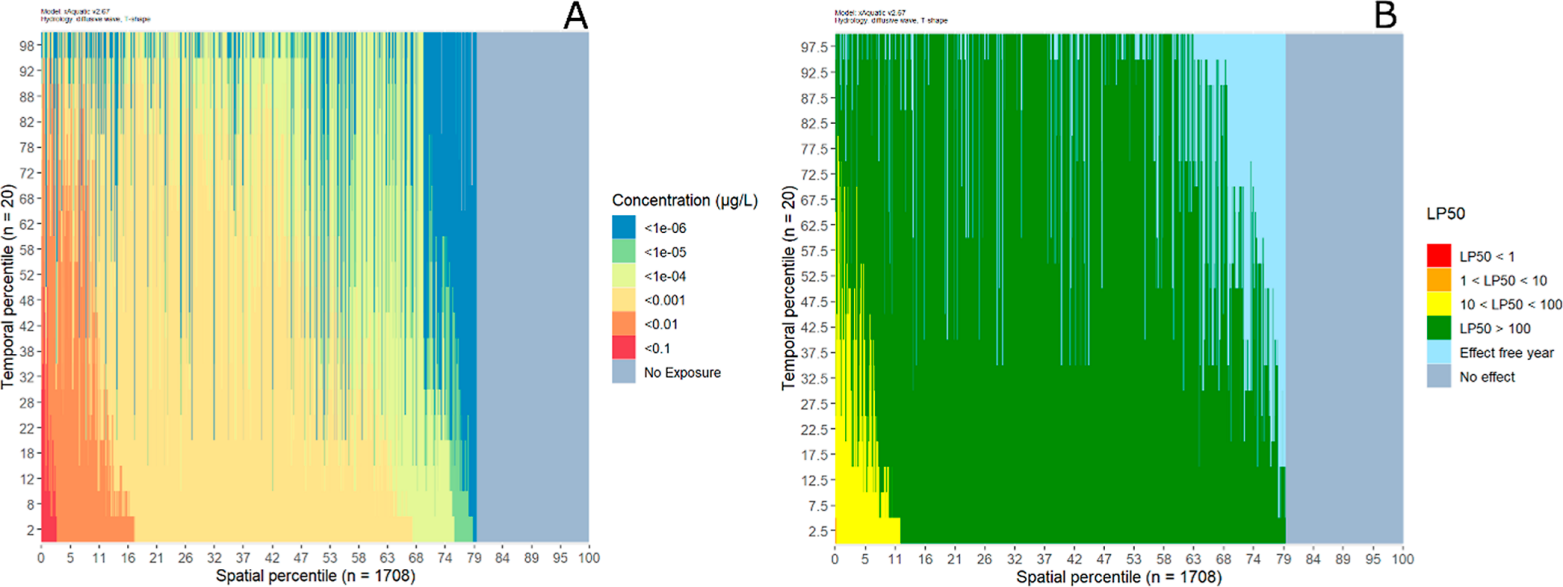
Spatiotemporal percentile plots of PEC_max_ surface water (A) and LP_50_ values (B). Columns represent individual reaches; rows represent the 20 years in the assessment period. First, PEC_max_ and LP_50_ are sorted within reaches in descending and ascending order, respectively (i.e., highest PEC_max_ and lowest LP_50_ at the bottom). Next, reaches are sorted along the *x*-axis such that the reaches with the highest PEC_max_ and lowest LP_50_ value in a year are located in the bottom left corner. No exposure (A) indicates reaches that have no exposure during the 20 year period; effect free year and no effect (B) indicate reaches where no fit of the LP_50_ value was possible or where no exposure occurs, respectively. Results were based on simulation with 75% drift reduction and a 10 m buffer as mitigation options.

To consider space and time simultaneously and explicitly, Figure 4A shows the spatiotemporally ranked annual maximum PEC values in surface water, following a similar approach to that proposed by Boesten.^3^ In the figure, each column represents a reach, and each row represents a year. There are 20 years in the simulation, and results are based on one simulation run. Hence, a single year covers 5% of the 20 year assessment period. First, within reaches, the years are sorted (in ascending order). Next, the reaches are sorted (in descending order) based on the reach that has the highest PEC_max_. This results in an ordering that generally decreases when moving from the bottom left corner to the top right corner, whereby the values along a row can represent different years. Note that in Boesten,^3^ the highest PEC values are in the top-right corner, whereas here, the highest PEC_max_ values are in the bottom-left corner. The figure shows what spatial percentile is above or below a certain PEC_max_ category per temporal percentile. In our case, for example, it shows that approximately 3% (51 out of 1708) of the reaches have PEC_max_ values between 0.1 and 0.01 μg/L for about 10% (2 out of 20) of the years. Conversely, in 97% (1657 out of 1708) of the reaches, PEC_max_ values are below 0.01 μg/L for about 90% (18 out of 20) of the years. Additionally, in the Rummen catchment, ca. 20% (ca. 342 out of 1708) of reaches are never exposed (directly nor indirectly) and may be considered as areas of no concern from a RA perspective.

Similarly, Figure 4B shows the spatiotemporally ranked LP_50_ values for the LGUTS-IT model for *A. aquaticus* (see the Supporting Information for results for *C. dipterum* and *G. pulex*). Reaches that do not receive any substance over the 20 year simulation period or at such low concentrations that no fit of the LGUTS model was possible are indicated in light blue and gray, respectively. The figure shows what spatial percentile is above/below a certain LP_50_ category per temporal percentile. For example, *A. aquaticus* has no reaches with LP_50_ values < 1, but approximately 10% (ca. 171 out of 1708) of reaches have values between 10 and 100 for not more than 5% (1 out of 20) of the years. Indeed, there are reaches with LP_50_ in the range between 10 and 100 for more than a single year, but they represent a smaller percentile of space. Additionally, there is a small percentage of reaches that have LP_50_ values between 1 and 10 for about 5% (1 out of 20) of the years. Such reaches are of higher concern and may benefit from targeted mitigation measures in addition to standard mitigation measures.

### 3.5 Leveraging Landscape-Level RA at Local Scales

**Figure 5:**
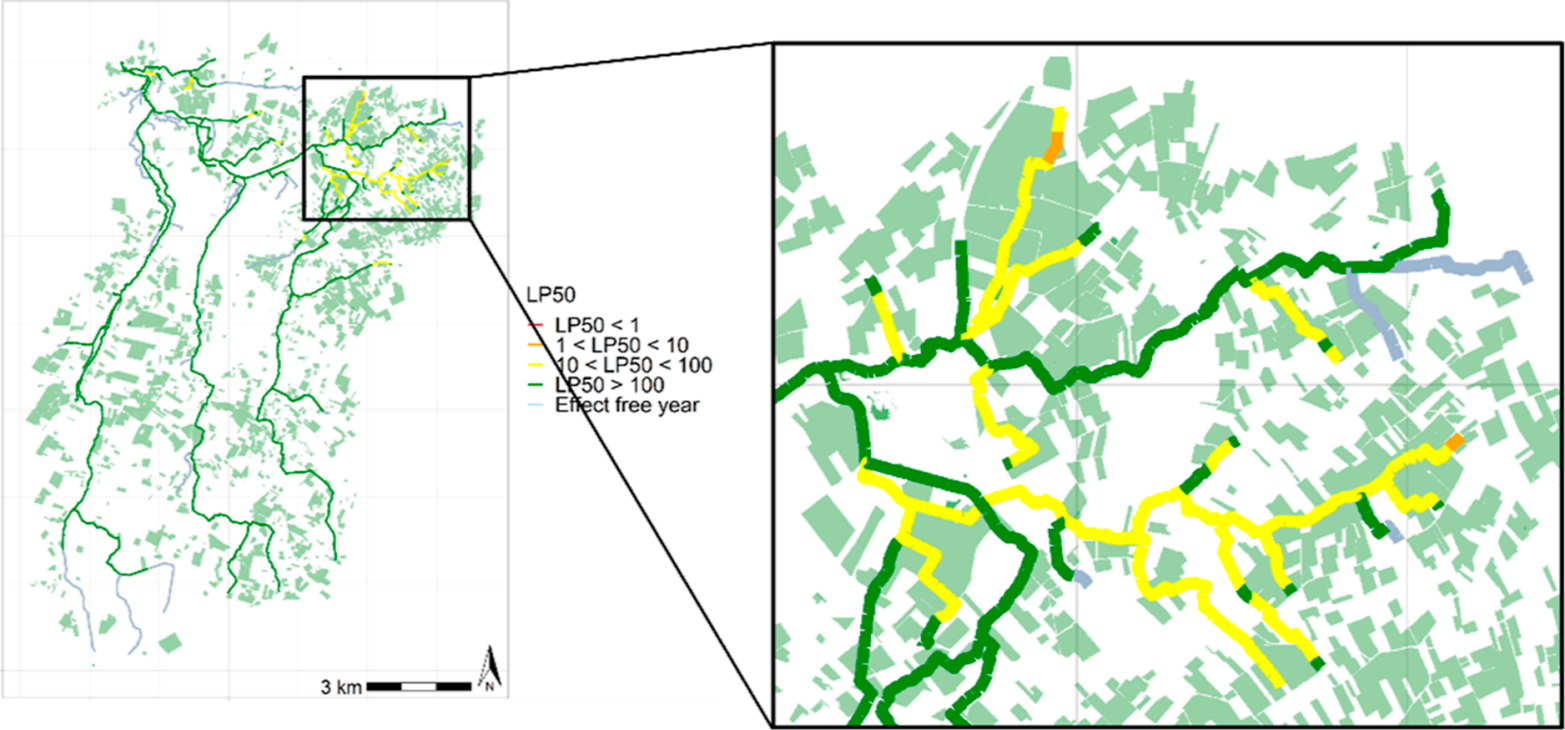
LP_50_ categories for the 5th percentile year in reaches in a 20 year assessment period; hence, for each reach, the worst year is displayed. The zoomed-in view shows that at local scales, the variation can be substantial.

Since the explicit spatiotemporal approach outlined in Section 3.4 retains information on variability over time and space (reaches), it is possible to, for example, create a spatial plot of the worst LP_50_ values (i.e., the fifth percentile year). An example for *A. aquaticus* is shown in Figure 5 (see Figures S13–S17 for results for *C. dipterum* and *G. pulex*). Note that each LP_50_ can represent a different year in this plot, i.e., all reaches show the LP_50_ category they get in the worst year in this example. The zoomed-in view clearly highlights reaches that are of concern with an LP_50_ category between 1 and 10. In such areas, targeted mitigation measures may be asked for by a regulator (in addition to the mitigation measures already in place). Through simulation with the xAquaticRisk model, the effects of such localized mitigation measures could be explored, allowing for a tailored RA process. Note that there are also several reaches (in gray, Figure 5) that fall within the “no effect” category, showing that the variation in risk levels can be substantial depending on the configuration of the local landscape. This information can inform the regulatory RA but also risk managers about environmental risk at local scales set within the broader context of landscape-scale RA.

### 3.6 Links to Current Risk Assessment Guidance

The EFSA PPR Panel^5^ proposes the specific protection goal for aquatic invertebrates to be based on their abundance and/or biomass in edge-of-field surface waters. Within the definition of the specific protection goal, there are two options for selecting the appropriate magnitude of the effect: the ecological threshold option (accepting only negligible effects on the population level) and the ecological recovery option (accepting some population level effects if recovery takes place within an acceptable time-window). In this study, only the ecological threshold option is relevant as we apply a TKTD model that does not allow for recovery (see ref (12)). The spatial scale in standard Tier 2C assessment is the edge-of-field scale. Likewise, for exposure assessments, the spatial unit that is considered is the edge-of-field water body.^5,9^ Translating this to the landscape-integrated model results presented in this study and considering the fruit orchards in the Rummen catchment as the area of intended use would imply that risks should be considered in all reaches that are located next to fruit orchards in the Rummen catchment. In our approach, however, reaches can be exposed directly (from spray-drift) or indirectly (from transport in the water) following applications on (any of) the fields in the catchment area. As such, it makes sense to consider not only the adjacent water courses as the “target” but all directly and indirectly exposed reaches as “target” in the RA. The precise effect level for the magnitude “negligible” is not clearly defined. Moreover, there is no guidance on how to translate this threshold from a single edge-of-field assessment to a landscape-scale assessment. At the edge-of-field scale, an effect may be considered unacceptable if the effects are larger than “negligible”, or concretely for this study if the LP_50_ is smaller than 100.^12^ Yet, at landscape scales, an effect may be considered “negligible” if the LP_50_ is larger than 100 in a predetermined spatiotemporal percentile, e.g., it must be at least 100 in at least 90% of reaches for at least 90% of the assessment period (i.e., 20 years). In future guidance on landscape-scale RA, a decision needs to be made on the appropriate/desired level of (spatiotemporal) effects and thus on the appropriate/desired level of aggregation to determine the relevant end points.

The potential benefits of an integrated and spatially explicit approach to RA have recently been recognized.^26^ Previous studies have developed such integrated fate and effect models for terrestrial systems, looking at, for example, earthworms,^27,28^ and at landscape scales for carabid beetles^29,30^ and vertebrates.^31−33^ In aquatic systems, studies have looked at the role of spatiotemporal dynamics in fate and effects using monitoring data,^34^ modeling,^14^ and ecotoxicological effects of pesticides on the ecosystem level using community trait data.^35^ In this study, we present a new landscape-scale model for aquatic systems and illustrate how model outputs can be interpreted in a RA context. This study provides new approaches to RA that can help to better understand and take account of the spatial and temporal variations common to complex systems and the nature of effects of pesticide use, which is a prerequisite to derive more tangible RA end points for higher tier RA. We presented several assessment end points with different levels of aggregation that may be used to address specific protection goals and inform risk assessors. The present study provides concrete options for the adoption of spatiotemporally explicit landscape-scale model results in the RA. However, dialogue within the community is needed on the eventual appropriate end points and the definition of representative landscape scenarios that can be used in landscape-scale RA.

The landscape-scale model, as presented here, includes environmental variability, but biological complexity (e.g., reproduction, migration, competition for resources, and space) is not considered with the LGUTS effect module (which simulates an arbitrary period of a year from the 1st of January to the 31st of December). As such, the options for landscape-scale RA using LGUTS are limited to addressing the ecological threshold option for the specific protection goal only. In future work, additional analyses will be performed by incorporating a population model into the already developed landscape model that was applied in this study. This means that a higher level of biological complexity will be considered, and with such a population model, aspects related to reproduction, recovery, metapopulation structure, and network connectivity can be incorporated into the assessment. Also, with a population model, the ecological recovery option for the specific protection goal can be considered, whereby populated reaches can serve as recolonization areas and refugia. Again, a dialogue will be necessary to decide the appropriate risk levels and suitable end points.

### 3.7 Limitations and Further Development

Due to the random nature of the drift deposition that is strongly dependent on the wind direction, the graphs presented here appear somewhat noisy (e.g., Figure 5). The timing of a drift event further affects the concentration in the watercourse and thereafter the effects on the considered species. Model outcomes would benefit from an approach to investigate the effect of application timing (both within and between years) and to use the ensemble of the model runs (from a Monte Carlo simulation, which will be an integral part of future experiments). For example, to examine the impact that the sampling of wind directions has at different spatial scales, a sensitivity analysis could be conducted, e.g., by comparing expectancy values derived from multiple Monte Carlo runs. Additionally, for some components such as LGUTS, uncertainty could be propagated by calculating confidence intervals on model outputs. Indeed, a full uncertainty analysis of the model was out of scope for this paper, given its proof-of-concept nature; however, this could be part of future experiments again using Monte Carlo simulations. Such insight into the uncertainties associated with the individual model components and overall model outputs may be helpful for both researchers and regulators, as it highlights which elements of the model may require further investigation or which components are particularly sensitive to changing the model outputs.

The current simulation spans a single 20 year period; thus, each year represents 5% of the total assessment period. With multiple Monte Carlo runs, the LP_50_ values calculated for a reach and for each Monte Carlo run can be “stacked” (owing to the independent nature of the LP_50_ values within the same reach as there is no carry-over effect from one year to the next). This would allow for the calculation of lower percentiles of time while also reducing the noise in the outputs. Currently, spray drift is the only entry route currently considered, whereas other transport pathways like runoff, erosion, and subsurface flow may also contribute to the total input of pesticide mass to surface waters. As this study is an example case study showcasing the xAquaticRisk model and a potential use of the model, ignoring these additional routes is deemed acceptable. However, for real-world cases, this may be insufficient, particularly for more mobile compounds. Therefore, in follow-up steps, additional exposure pathways such as emission via drain flow and runoff will be added as components to the modular landscape model, assuming more realistic uses of one substance and also including exposure to multiple substances due to pesticide uses in the entire landscape.

Lastly, the xAquaticRisk model presented here is publicly available via GitHub, allowing researchers to set up the model for their own research needs. Moreover, model components can be readily created by the research community, which is greatly facilitated by the modular nature of the model and the flexibility of being able to accommodate a multitude of programming languages, thus giving the xAquaticRisk model a potential scope for use that goes well beyond the example case-study presented here.

## Supporting Information Available

Schematic overview of the xAquaticRisk model; all GUTS model fits; additional substance properties; hydrogeographic information; drift deposition information; and supplementary model output results (PDF)
