# Supplementary material for: Aquatic Risks at the Landscape Scale: A Case Study for Pyrethroid Use in Pome Fruit Orchards in Belgium
Source: Environ Sci Technol. 2023 Oct 5;57(41):15608–16. doi: 10.1021/acs.est.3c02716 (PMC10586366; doi:10.1021/acs.est.3c02716)
Supplement: Supplementary file 1 — es3c02716_si_001.pdf [file es3c02716_si_001.pdf]

Supporting Information

Manuscript title:

Aquatic risks at landscape scale: a case study for pyrethroid use in pome fruit orchards in Belgium

Authors: Willem B. Buddendorf, Louise Wipfler, Wim Beltman, Hans Baveco, Maarten C. Braakhekke, Sascha Bub, André Gergs, Thorsten Schad.

Number of pages: 23

Number of figures: 18

Number of tables: 6

Schematic overview of the xAquaticRisk model

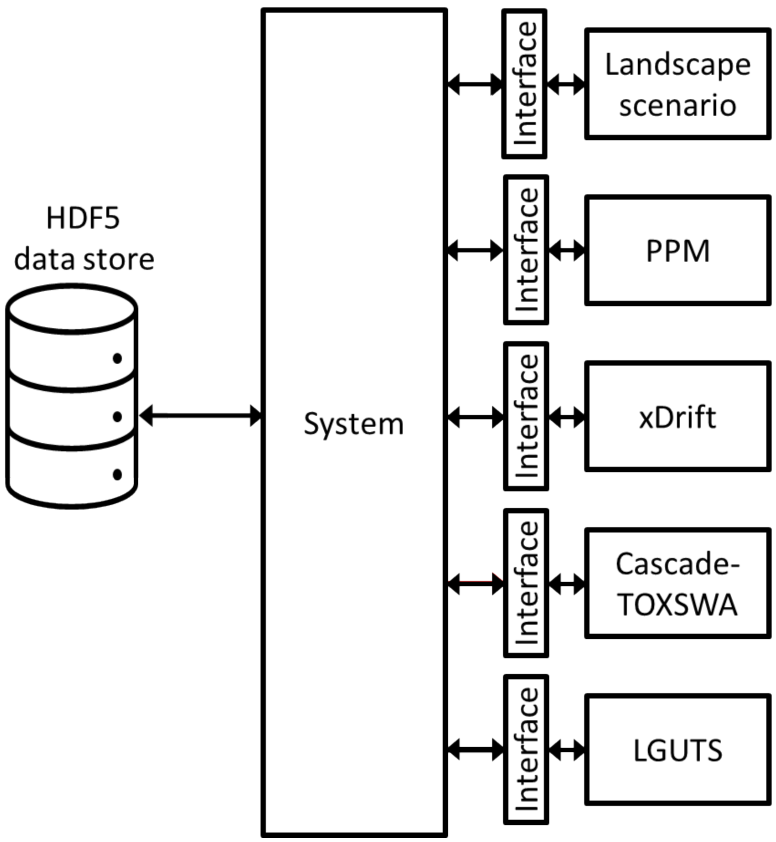

Figure S0. Schematic overview of the xAquaticRisk model and its components (see https://github.com/xlandscape/xAquaticRisk/tree/2.67). All data is stored centrally in the HDF5 data store, the model system handles all I/O processes between the datastore and the individual components. Model components can be run in sequence (from top to bottom) or as stand-alone components provided the required input data is available in the data store. The landscape scenario contains all geo-information, the hydrology, meteorological data, and other information relevant to

the specific scenario that is used. The landscape scenario is processed by the model system and data is stored in the HDF5 data store. The Plant Protection Measure (PPM) component handles agricultural management and generates input data on application characteristics: location, timing, application rate, and used equipment. The xDrift component simulates spray drift deposition. CASCADE_TOXSWA simulates pesticide fate in interconnected water courses. LGUTS simulates pesticide effects, using time series of simulated pesticide concentrations.

Peak concentrations in Rummen catchment in 1998

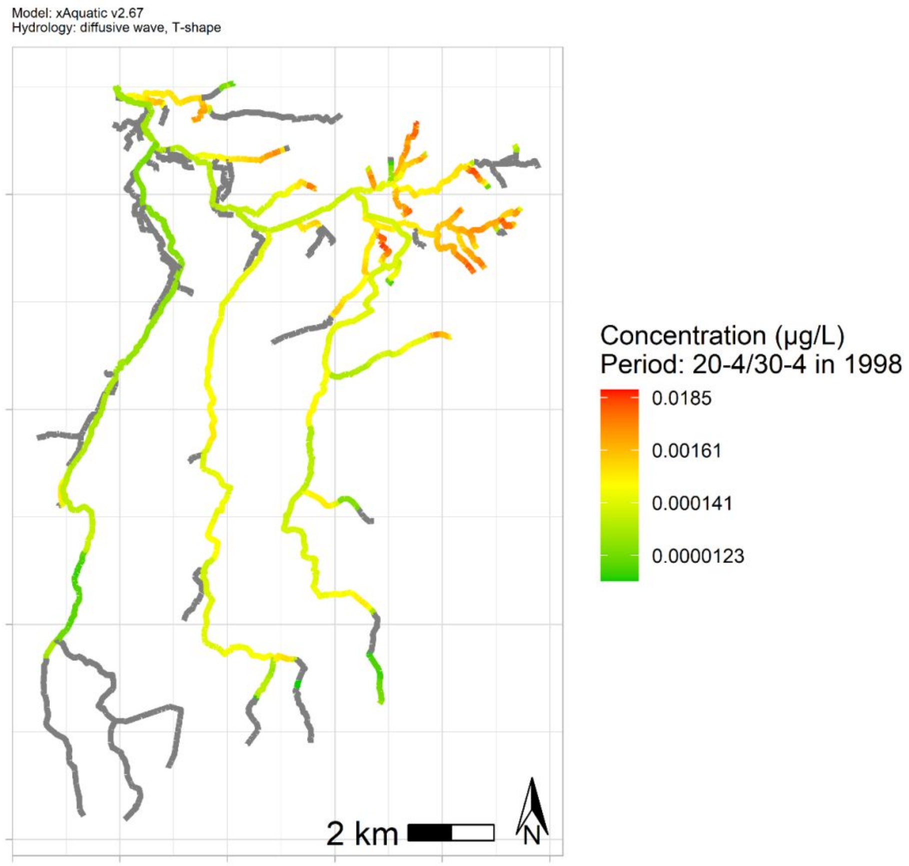

Figure S1. Maximum predicted environmental concentrations in the Rummen catchment during the application window for the year 1998. Highest concentrations are predicted in the northeast of the catchment.

Parameterisation of GUTS

In this study the ‘reduced GUTS’ models for individual tolerance and stochastic death model versions were fitted (Jager et al., 2011; Jager and Ashauer, 2018). Below, GUTS-RED-IT refers to reduced GUTS for the individual tolerance model; GUTS-RED-SD refers to reduced GUTS for the stochastic death model.

Table S1. GUTS parameters for three aquatic species *Asellus aquaticus*, *Cloeon dipterum*, and *Gammarus pulex* used in the LGUTS simulation at landscape scale, for model fits and report details see figures S2-S7 below. Note that the background mortality derived from the fits were not used in LGUTS simulations and are thus not listed. Details on laboratory studies used for model parametrisations are provided in study reports which are available on request by sending an email to cropscience-transparency@bayer.com referring to the study report number.

GUTS	Parameter	*Asellus aquaticus*	*Cloeon dipterum*	*Gammarus pulex*
IT	kd [h-1]	0.0005541	0.01416	0.002324
	alpha [ng/L]	0.4429	36.27	0.8206
	beta [-]	1.582	1.648	3.049
SD	kd [h-1]	0.01188	0.03719	0.008858

z [ng/L]	0.05507	4.702	0.6292
kk [L ng-1 h-1]	0.002615	0.0009361	0.01989

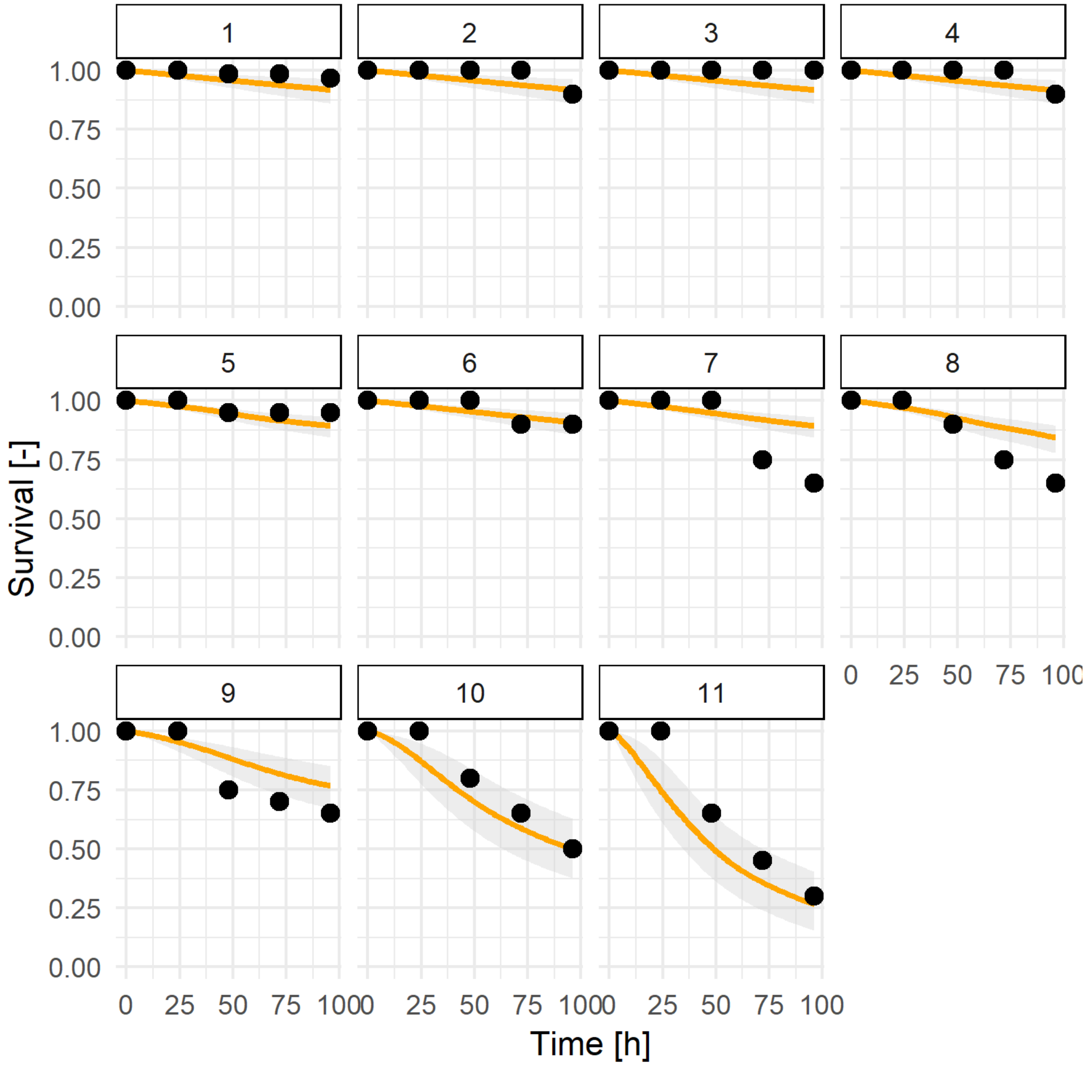

Figure S2. *Asellus aquaticus* survival predictions using the GUTS-RED-IT assumption, calibrated on data from study M-199681-01-2 (Bayer AG, 2001). Plotted are the model predictions (orange

lines) and observed data (black dots) for 11 initial concentrations: 1: 0 ng/L, 2: 0.03 ng/L, 3: 0.06 ng/L, 4: 0.13 ng/L, 5: 0.31 ng/L, 6: 0.46 ng/L, 7: 0.79 ng/L, 8: 1.85 ng/L, 9: 2.95 ng/L, 10: 7.58 ng/L, 11:16.6 ng/L. Grey areas represent the 95% confidence interval.

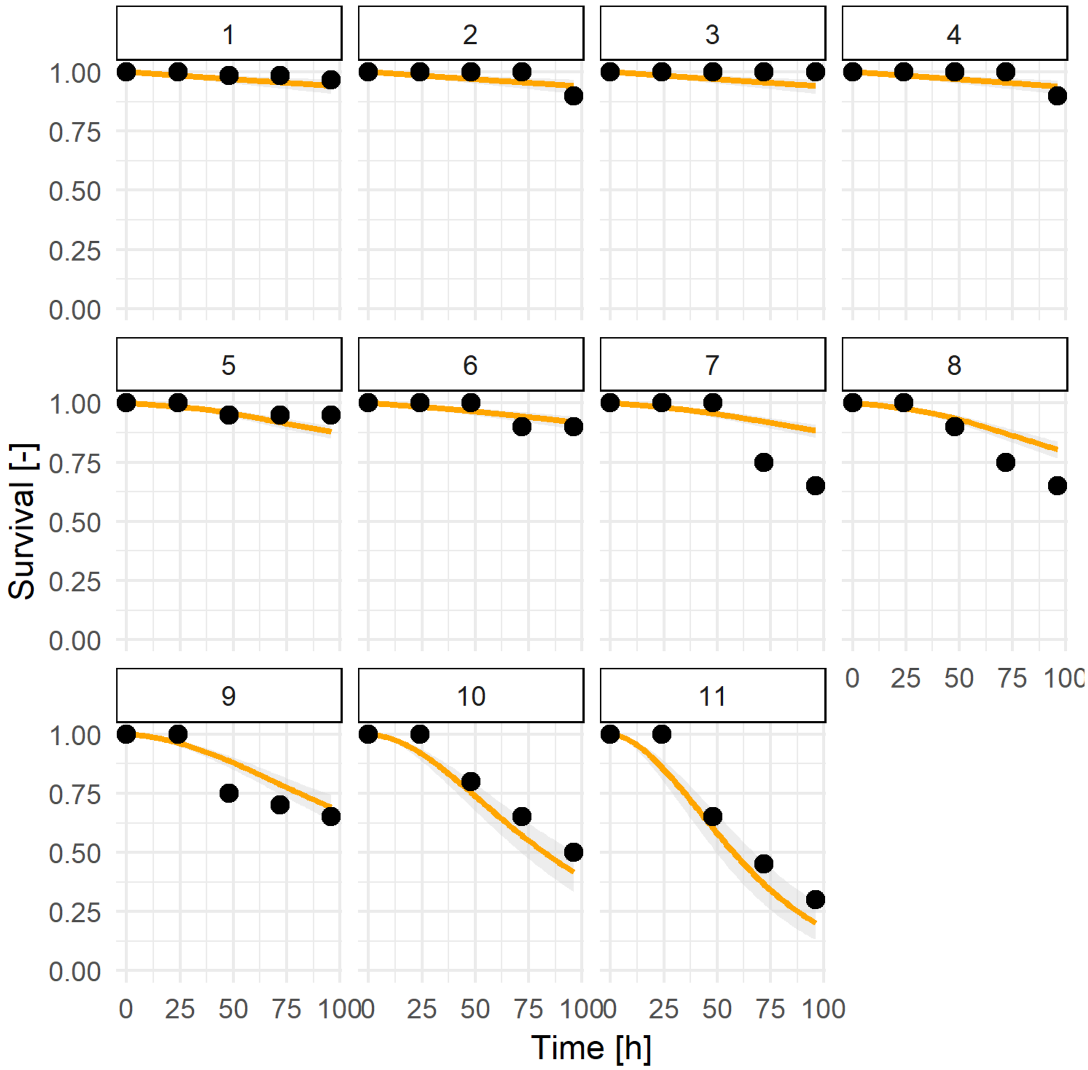

Figure S3. *Asellus aquaticus* survival predictions using the GUTS-RED-SD assumption, calibrated on data from study M-199681-01-2. Plotted are the model predictions (orange lines) and observed data (black dots) for 11 initial concentrations: 1: 0 ng/L, 2: 0.03 ng/L, 3: 0.06 ng/L, 4: 0.13 ng/L, 5: 0.31 ng/L, 6: 0.46 ng/L, 7: 0.79 ng/L, 8: 1.85 ng/L, 9: 2.95 ng/L, 10: 7.58 ng/L, 11:16.6 ng/L. Grey areas represent the 95% confidence interval.

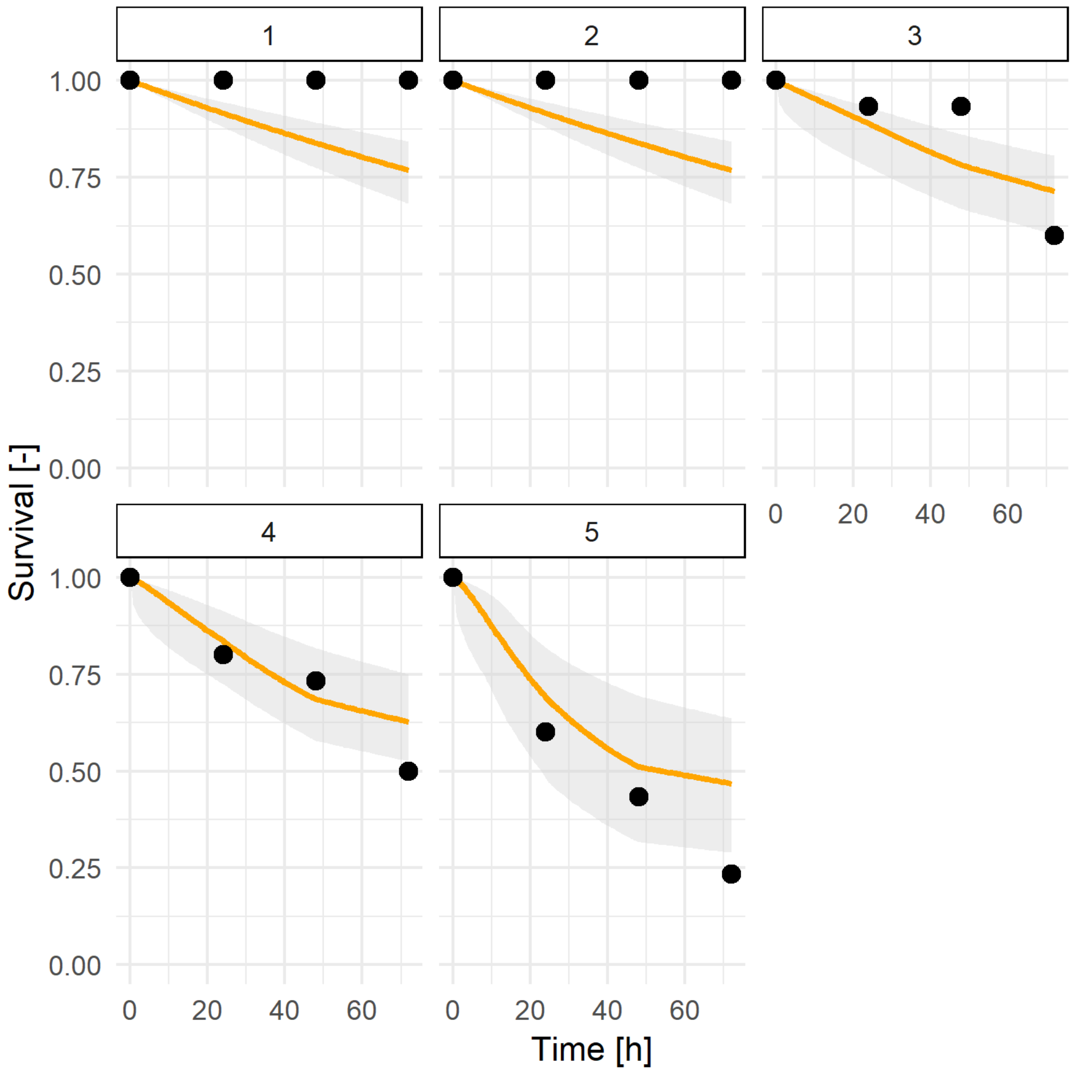

Figure S4. *Cloeon dipterum* survival predictions using the GUTS-RED-IT assumption, calibrated on data from study M-643155-01-1 (Bayer AG, 2018a). Plotted are the model predictions (orange lines) and observed data (black dots) for: 1: control, 2: solvent control, 3: 20 ng/L, 4: 40 ng/L, 5: 80 ng/L. Grey areas represent the 95% confidence interval.

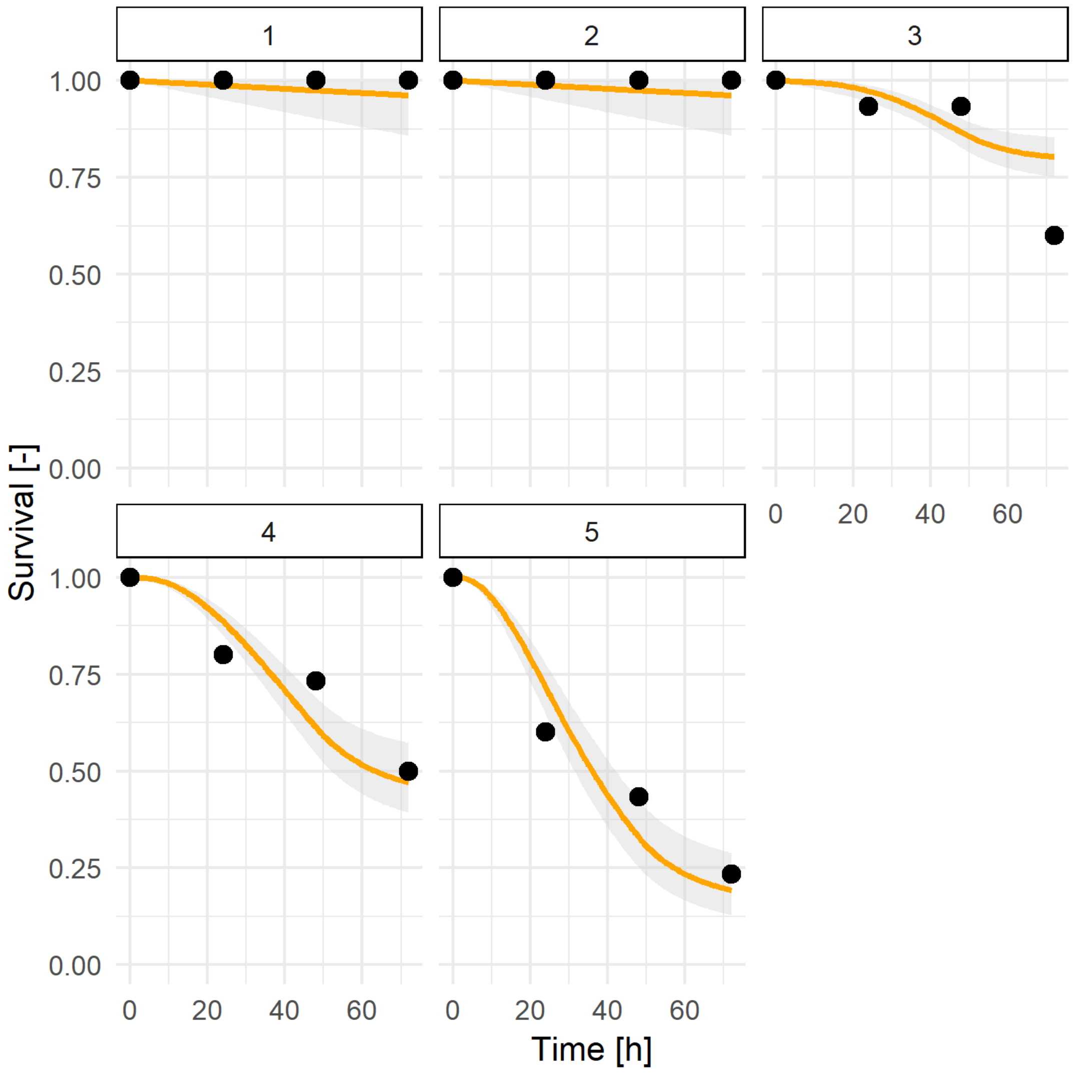

Figure S5. *Cloeon dipterum* survival predictions using the GUTS-RED-SD assumption, calibrated on data from study M-643155-01-1. Plotted are the model predictions (orange lines) and observed data (black dots) for: 1: control, 2: solvent control, 3: 20 ng/L, 4: 40 ng/L, 5: 80 ng/L. Grey areas represent the 95% confidence interval.

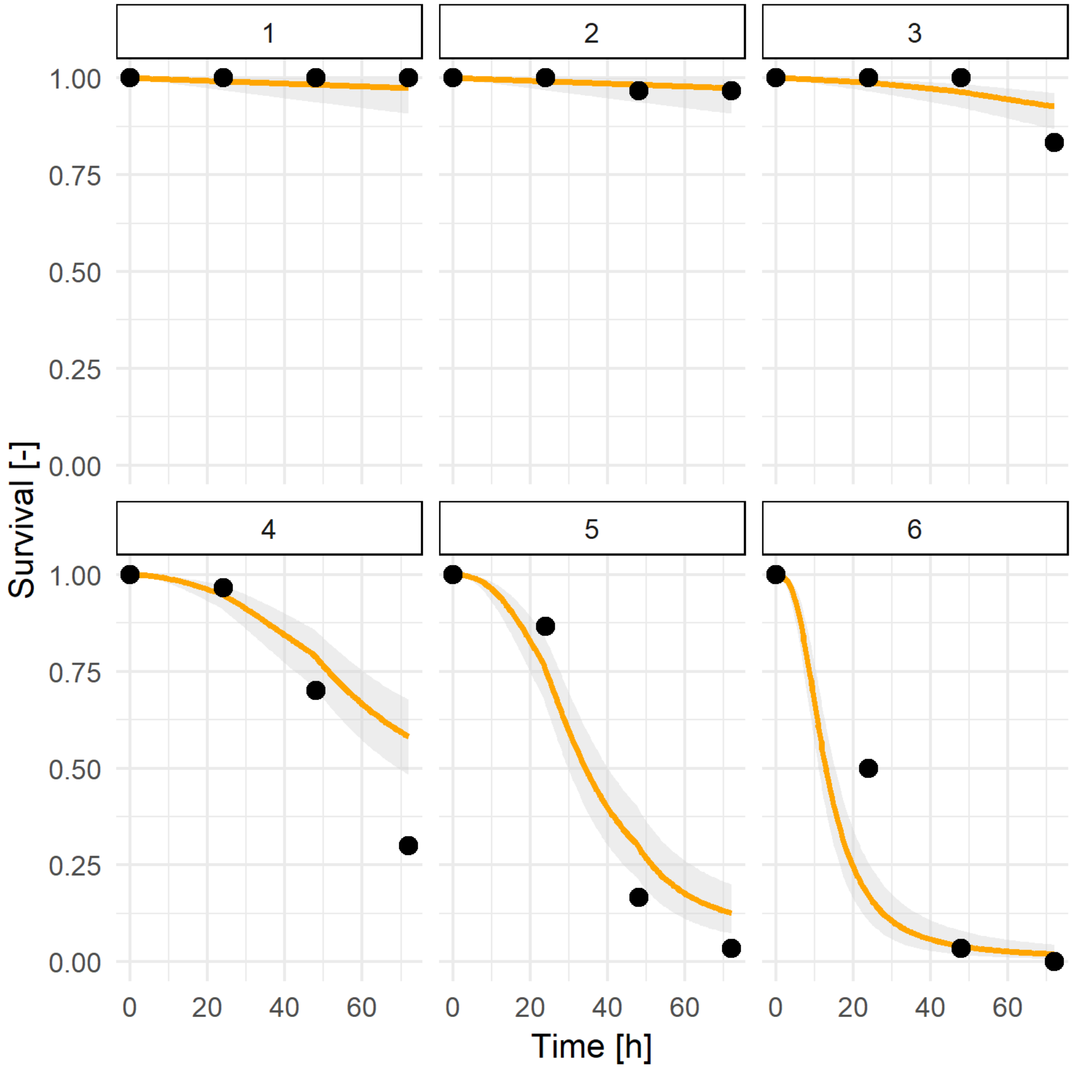

Figure S6. *Gammarus pulex* survival predictions using the GUTS-RED-IT assumption, calibrated on data from study M-643326-01-1. Plotted are the model predictions (orange lines) and observed

data (black dots) for six treatments: 1: Control, 2: Solvent Control, 3: 3.5 ng/L, 4: 7 ng/L, 5: 14 ng/L, 6: 28 ng/L (nominal). Grey areas represent the 95% confidence interval.

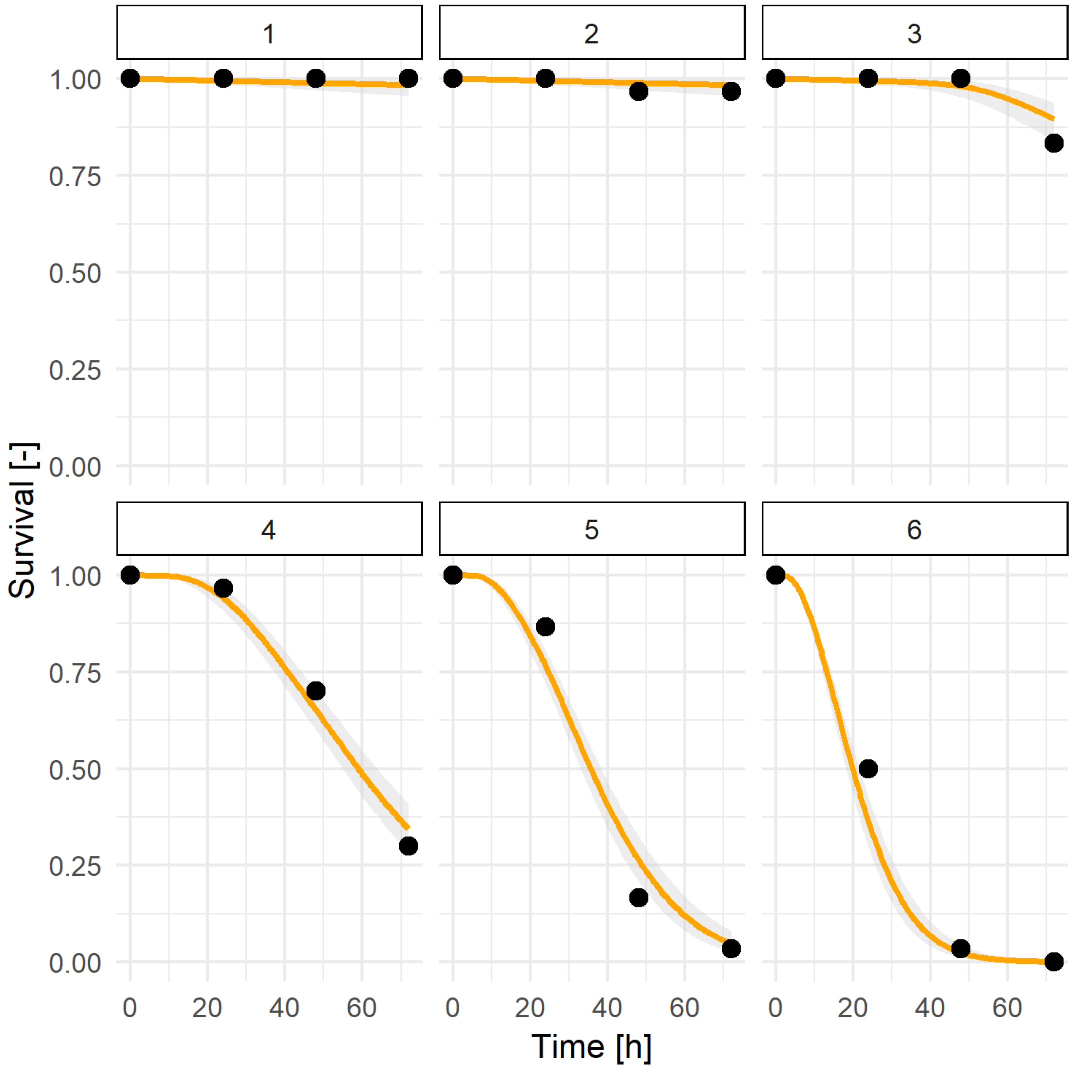

Figure S7. *Gammarus pulex* survival predictions using the GUTS-RED-SD assumption, calibrated on data from study M-643326-01-1 (Bayer AG, 2018b). Plotted are the model predictions (orange

lines) and observed data (black dots) for six treatments: 1: Control, 2: Solvent Control, 3: 3.5 ng/L, 4: 7 ng/L, 5: 14 ng/L, 6: 28 ng/L (nominal). Grey areas represent the 95% confidence interval.

Table S2. Substance properties

Parameter	Units	Value
Molar mass	$g.mol^{-1}$	505.2
Saturated vapour pressure	Pa	1.10^{-6}
Reference temperature for saturated vapour pressure	C	20
Molar enthalpy of vaporization	$kJ.mol^{-1}$	95
Water solubility	mg.L-1	0.001
Reference temperature for water solubility	C	20
Molar enthalpy of dissolution	$kJ.mol^{-1}$	27
Reference diffusion coefficient in water	$m^2.d^{-1}$	$4.3.10^{-5}$
Half-life transformation in water at reference temperature	d	1000
Reference temperature for half-life measured in water	C	20
Molar activation enthalpy of transformation in water	$kJ.mol^{-1}$	65.4
Half-life transformation in sediment at reference temperature	d	43.9
Reference temperature for half-life in sediment	C	20

Molar activation enthalpy of transformation in sediment	$kJ.mol^{-1}$	65.4
Freundlich coefficient of equilibrium sorption for sediment	$L.kg^{-1}$	266821.3
Reference concentration in liquid phase for Freundlich coefficient for sediment	$mg.L^{-1}$	1
Freundlich exponent in sediment	-	0.93
Freundlich coefficient of equilibrium sorption for suspended solids	$L.kg^{-1}$	266821.3
Reference concentration in liquid phase for Freundlich sorption coefficient for suspended solids	$mg.L^{-1}$	1
Freundlich exponent suspended solids	-	0.93
Coefficient for linear sorption on macrophytes	$L.kg^{-1}$	0

Table S3. Hydrogeographic characteristics of reaches in Rummen catchment per Strahler order category for the 20 year evaluation period.

Strahler Order	Number of reaches	total Length (km)	bottom width (m)	Bank slope (hor:ver)	Channel depth (m)	Bank width (m)	Median water depth (m)	Manning n
1	934	79.5	0.5	1	1.25	1.25	0.08	0.035
2	563	48.2	1	1	1.5	1.5	0.19	0.027
3	107	9.4	2	0.5	2	2	0.27	0.018

4	104	8.9	4	0.5	2	2	0.22	0.010

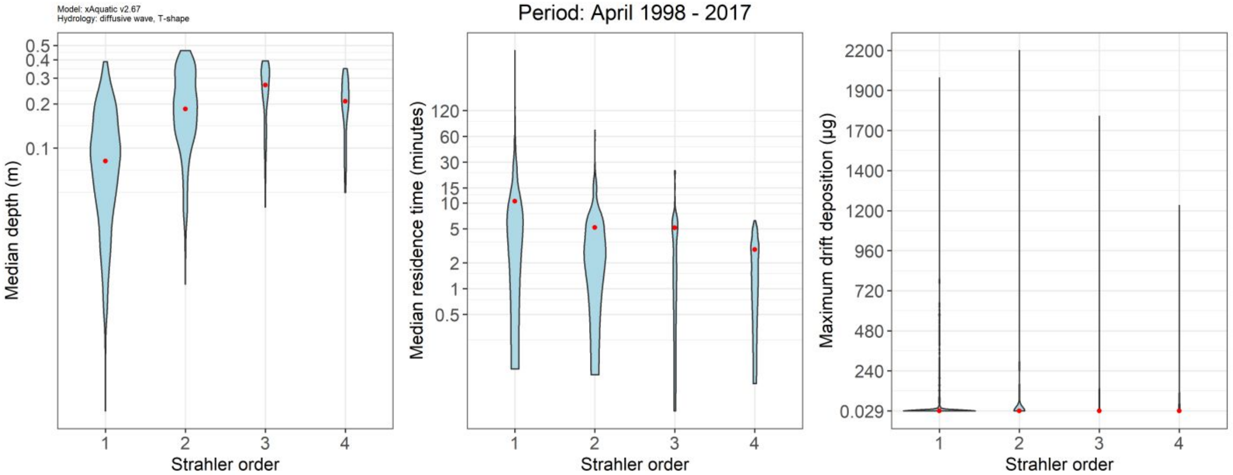

Figure S8. Violin plots showing the spread in median hourly water depths (left), median residence times in minutes (middle), and the maximum drift deposition (right) in reaches given per Strahler order for the period 20-30 April and for the full 20-year evaluation period. Area of the violin is scaled proportionally to the number of observations; red dots represent the median of the distributions.

Table S4. Percentage of reaches, per Strahler order, receiving substance from drift deposition or transfer from upstream reaches.

Strahler order	Number of reaches	Exposure to substance from loading drift and transfer (%)	Exposure to substance from transfer only (%)	Receiving no substance (%)
1	934	62	24.7	38
2	563	98.4	60	1.6
3	107	100	89.7	0
4	104	100	87.5	0

LGUTS IT model outputs

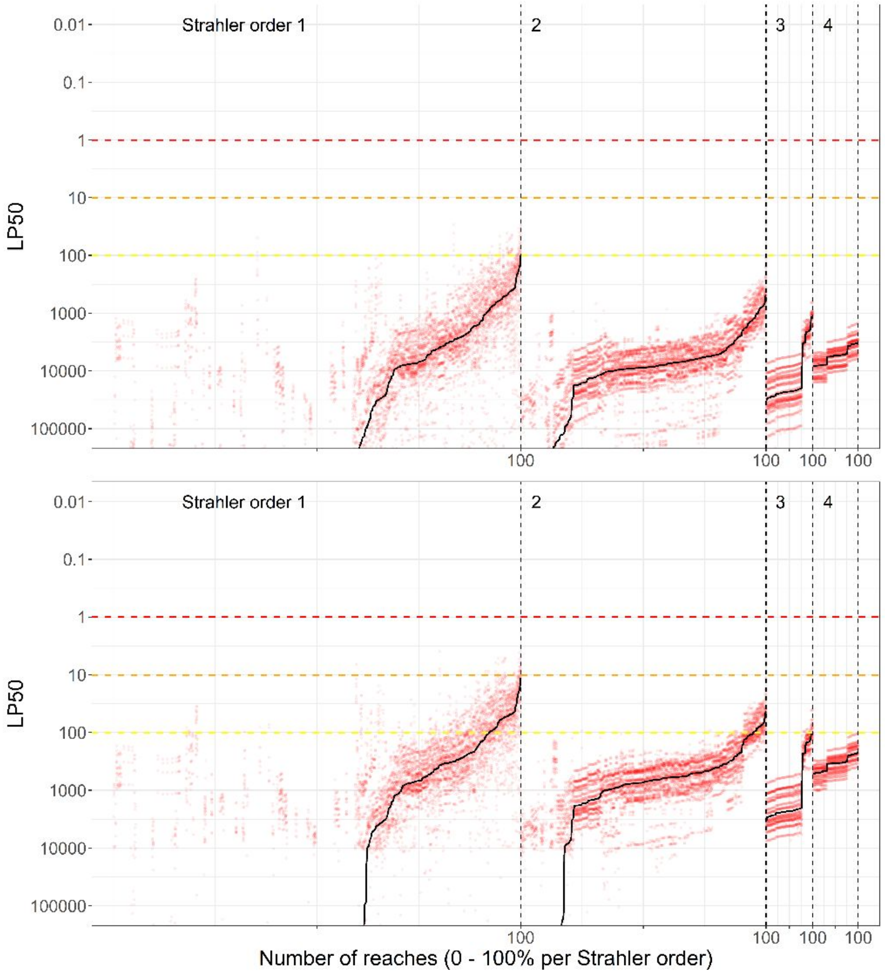

Figure S9. LP_{50} values calculate with the individual tolerance TKTD model for *Cloeon dipterum*, and *Gammarus pulex* per Strahler order. Reaches were sorted by their median along the x-axis. The y-axis was cut off at an LP_{50} value of 10^5.

Table S5. Range of LP_{50} values for the LGUTS-IT model for Cloeon dipterum and Gammarus pulex. Values of Inf are the result of reaches with no exposure and no subsequent effects.

Strahler order	*Asellus aquaticus*	*Cloeon dipterum*	*Gammarus pulex*
1	7.07 - Inf	29.43 - Inf	3.38 - Inf
2	24.59 - Inf	109.39 - Inf	11.44 - Inf
3	74.26 – 14745.36	638.33 – 213868.7	40.2 – 7562.78
4	192.76 – 1850.37	1492.75 – 15824.87	103.61 – 1012.28

LGUTS SD model outputs

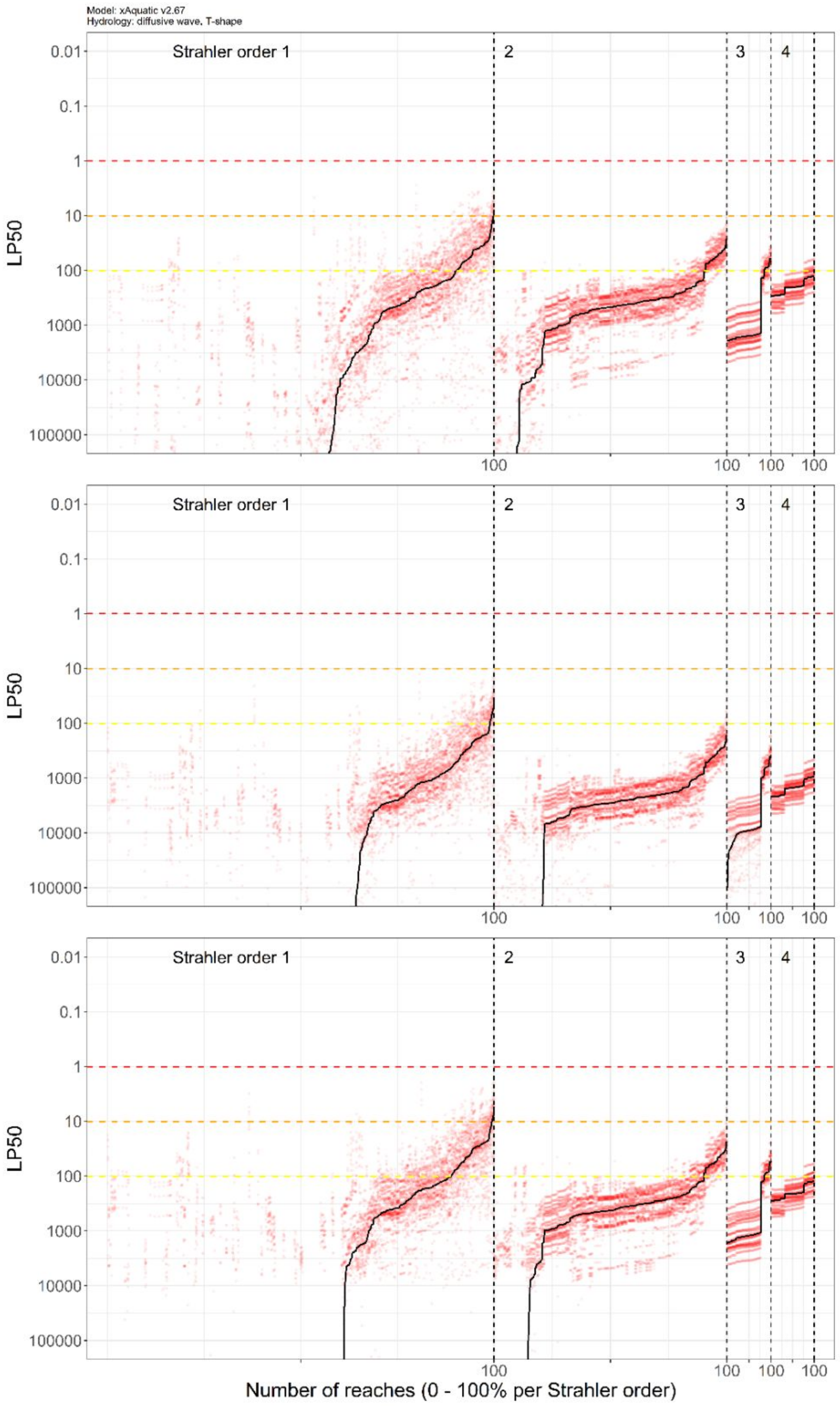
Model: xAquatic v2.67
Hydrology: diffusive wave, T-shape
Strahler order 1
2
3
4
LP50
0.01
0.1
1
10
100
1000
10000
100000
100
Number of reaches (0 - 100% per Strahler order)

Figure S10. LP_{50} values calculate with the stochastic death TKTD model for A. aquaticus, C. dipterum, and G. pulex per Strahler order. Reaches were sorted by their median along the x-axis. The y-axis was cut off at an LP_{50} value of 10^5.

Table S6. Range of LP_{50} values for the LGUTS-SD model for Asellus aquaticus, Cloeon dipterum, and Gammarus pulex. Values of Inf are the result of reaches with no exposure and no subsequent effects.

Strahler order	*Asellus aquaticus*	*Cloeon dipterum*	*Gammarus pulex*
1	2.30 - Inf	11.71 - Inf	1.96 - Inf
2	8.22 - Inf	39.78 - Inf	5.74 - Inf
3	23.93 – 4727.1	170.54 – Inf	24.49 – 4113.67
4	62.55 – 608.39	462.55 – 4460.89	60.23 – 555.89

Spatiotemporal GUTS-IT LP50 plots

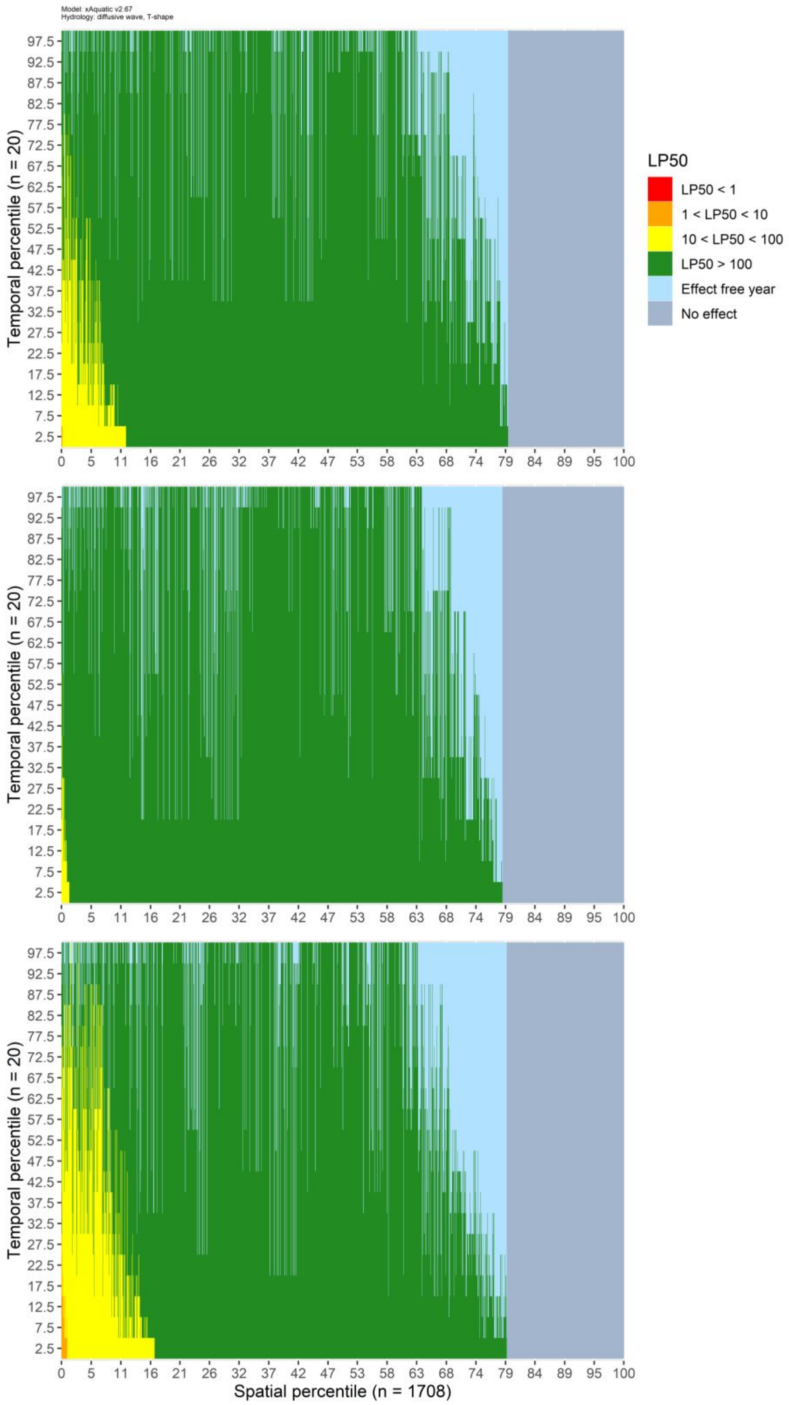

Figure S11. Spatiotemporal percentile plots of LP50 values for the GUTS-IT model for (from top to bottom): *Asellus aquaticus*, *Cloeon dipterum*, *Gammarus pulex*. Within each reach (columns)

values are ranked from low to high. Next, reaches were ranked according to the reach which had the the lowest LP50 in the first year (i.e., the 5%-ile year). Effect free year and No effect indicate reaches where no fit of the LP50 value was possible or where no exposure occurs, respectively. Results based on simulation with 75% drift reduction and a 10 m buffer as mitigation options.

Spatiotemporal GUTS-SD LP50 plots

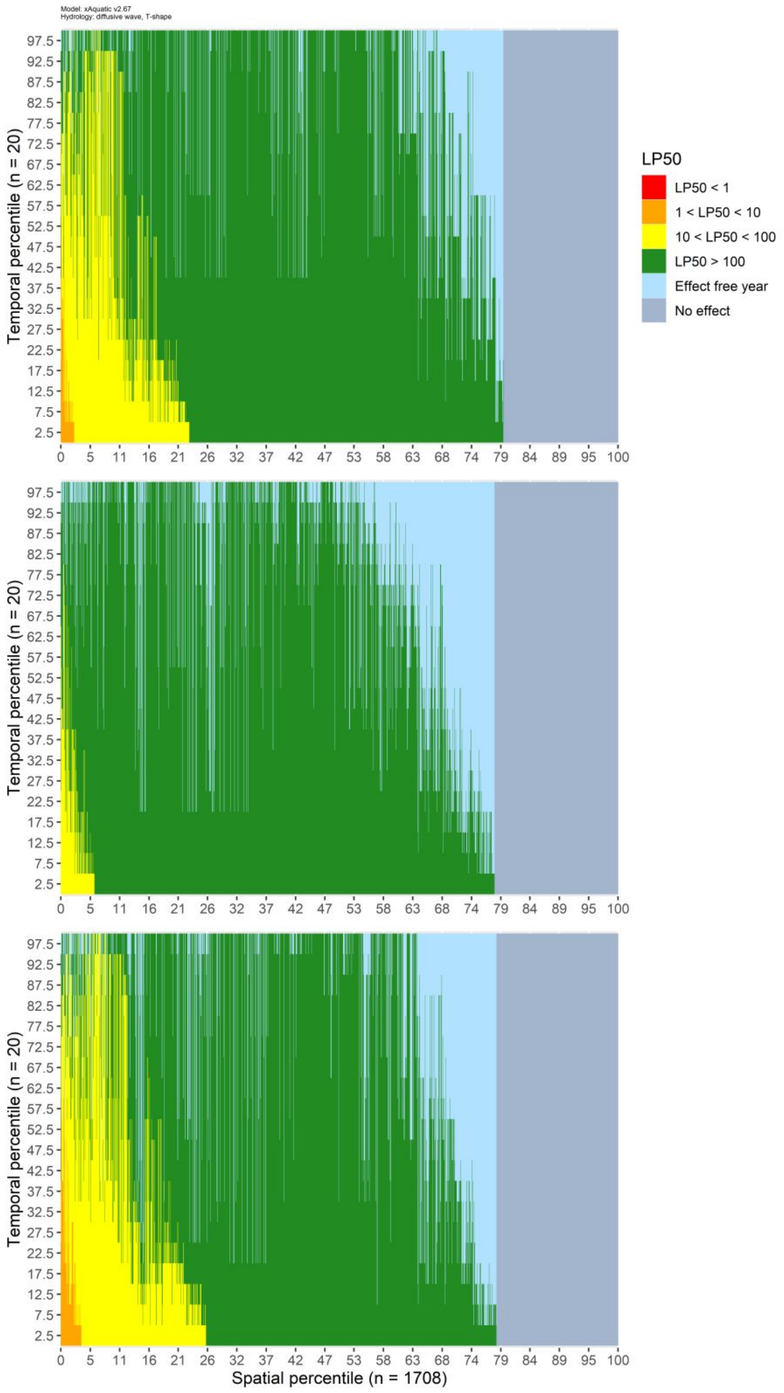

Figure S12. Spatiotemporal percentile plots of LP50 values for the GUTS-SD model for (top to bottom): *Asellus aquaticus, Cloeon dipterum*, and *Gammarus pulex*. Within each reach (columns)

values are ranked from low to high. Next, reaches were ranked according to the reach which had the the lowest LP50 in the first year (i.e., the 5%-ile year). Effect free year and No effect indicate reaches where no fit of the LP50 value was possible or where no exposure occurs, respectively. Results based on simulation with 75% drift reduction and a 10 m buffer as mitigation options.

Leveraging landscape scale RA at local scales (GUTS-IT model)

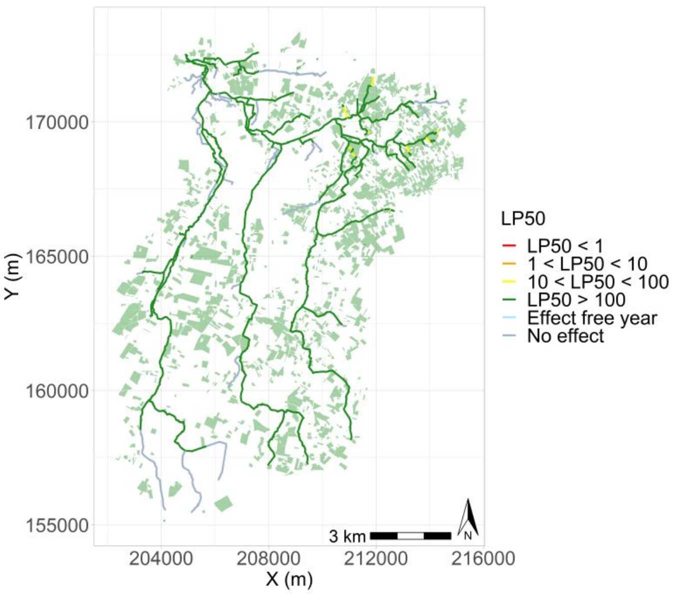

Figure S13. LP50 categories for *Cloeon dipterum* for the GUTS-IT model for the 5th percentile year in reaches in a 20-year assessment period, hence for each reach the worst year is displayed.

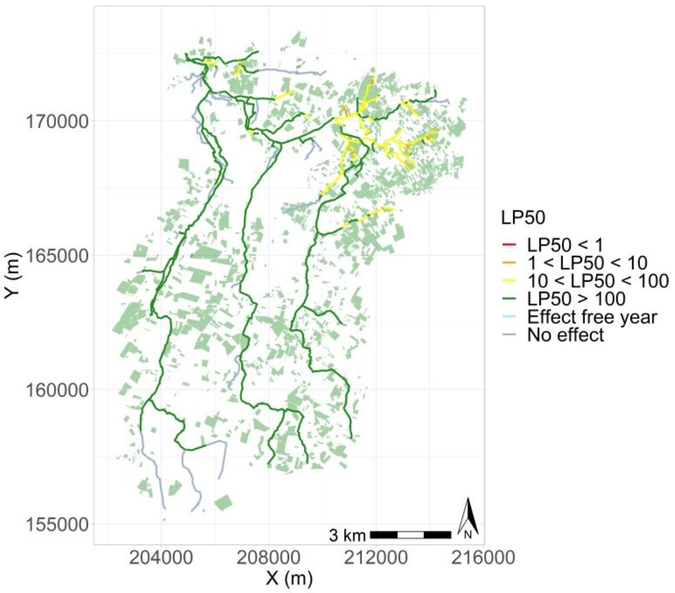

Figure S14. LP50 categories for *Gammarus pulex* for the GUTS-IT model for the 5th percentile year in reaches in a 20-year assessment period, hence for each reach the worst year is displayed.

Leveraging landscape scale RA at local scales (GUTS-SD model)

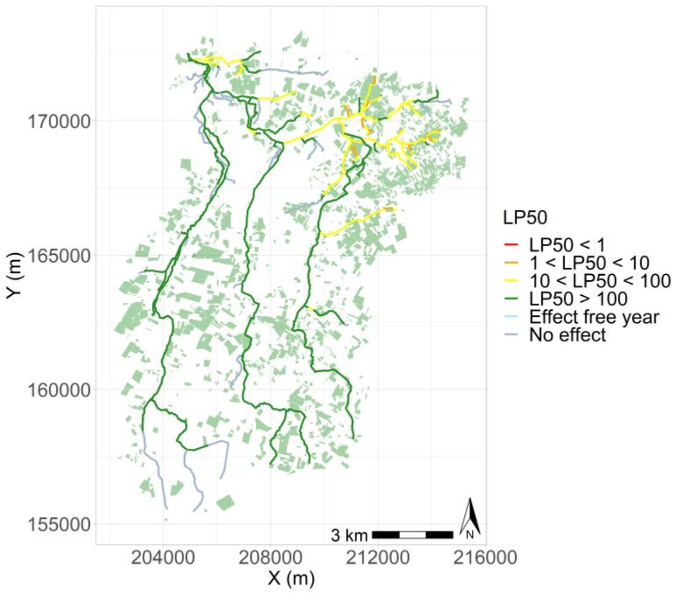

Figure S15. LP50 categories for *Asellus aquaticus* for the GUTS-SD model for the 5th percentile year in reaches in a 20-year assessment period, hence for each reach the worst year is displayed.

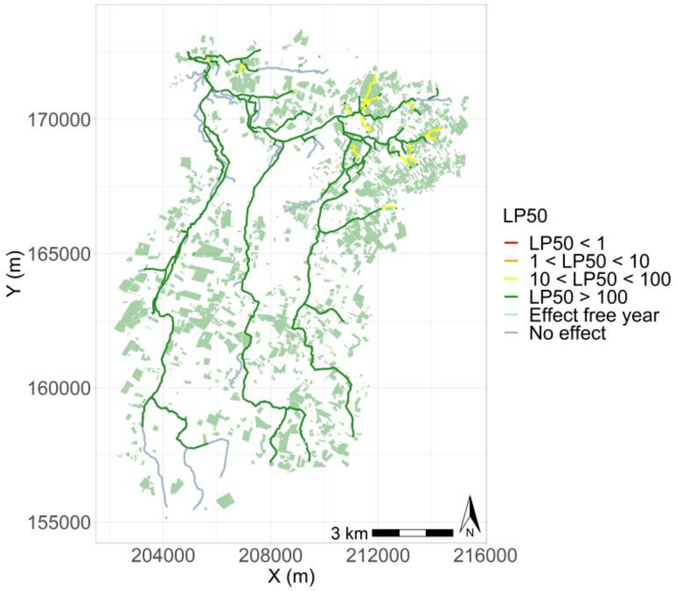

Figure S16. LP50 categories for *Cloeon dipterum* for the GUTS-SD model for the 5th percentile year in reaches in a 20-year assessment period, hence for each reach the worst year is displayed.

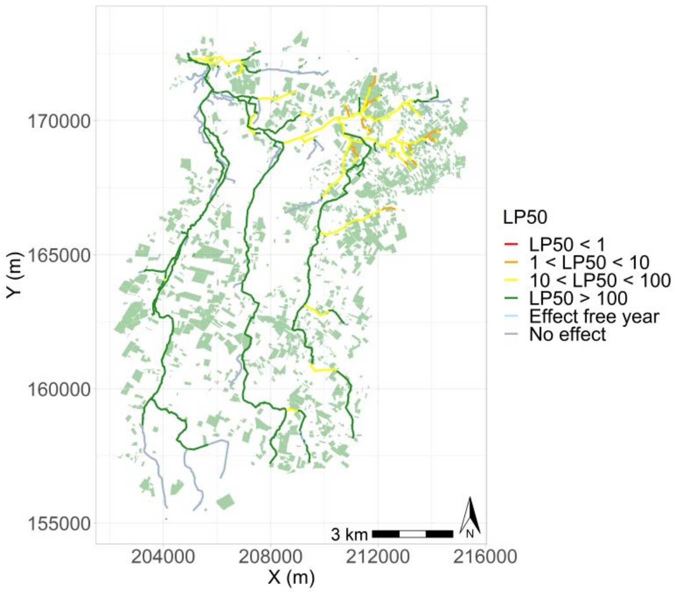

Figure S17. LP50 categories for *Gammarus pulex* for the GUTS-SD model for the 5th percentile year in reaches in a 20-year assessment period, hence for each reach the worst year is displayed.
